# Microbial Metabolites Orchestrate a Distinct Multi-Tiered Regulatory Network in the Intestinal Epithelium That Directs P-Glycoprotein Expression

**DOI:** 10.1128/mbio.01993-22

**Published:** 2022-08-15

**Authors:** Sage E. Foley, Michael J. Dente, Xuqiu Lei, Benjamin F. Sallis, Ethan B. Loew, Mario Meza-Segura, Katherine A. Fitzgerald, Beth A. McCormick

**Affiliations:** a Department of Microbiology and Physiological Systems, University of Massachusetts Chan Medical School, Worcester, Massachusetts, USA; b Program in Microbiome Dynamics, University of Massachusetts Chan Medical School, Worcester, Massachusetts, USA; c T.H. Chan School of Medicine, University of Massachusetts Chan Medical School, Worcester, Massachusetts, USA; d Program in Innate Immunity, Department of Medicine, University of Massachusetts Chan Medical School, Worcester, Massachusetts, USA; UT Southwestern Medical Center

**Keywords:** P-glycoprotein, multi-drug resistance transporter, microbiome, metabolites, butyrate, short-chain fatty acids, secondary bile acids, RNAseq, intestinal epithelium, inflammation

## Abstract

P-glycoprotein (P-gp) is a key component of the intestinal epithelium playing a pivotal role in removal of toxins and efflux of endocannabinoids to prevent excessive inflammation and sustain homeostasis. Recent studies revealed butyrate and secondary bile acids, produced by the intestinal microbiome, potentiate the induction of functional P-gp expression. We now aim to determine the molecular mechanism by which this functional microbiome output regulates P-gp. RNA sequencing of intestinal epithelial cells responding to butyrate and secondary bile acids in combination discovered a unique transcriptional program involving multiple pathways that converge on P-gp induction. Using shRNA knockdown and CRISPR/Cas9 knockout cell lines, as well as mouse models, we confirmed the RNA sequencing findings and discovered a role for intestinal HNF4α in P-gp regulation. These findings shed light on a sophisticated signaling network directed by intestinal microbial metabolites that orchestrate P-gp expression and highlight unappreciated connections between multiple pathways linked to colonic health.

## INTRODUCTION

P-glycoprotein (P-gp) is a highly conserved ATP-binding cassette (ABC) transporter with many roles beneficial to the mammalian host. Classically known as a multi-drug resistance transporter, P-gp protects cells via efflux of toxins and xenobiotics, as well as by exporting anti-inflammatory molecules to suppress aberrant neutrophil infiltration (1–3). Despite over a decade of knowledge that P-gp plays a central role in intestinal homeostasis, the precise molecular mechanism of its regulation in the intestine is poorly understood. We therefore sought to determine the intestinal epithelial cell (IEC) signaling networks that promote and maintain P-gp expression in the healthy colon.

P-gp is a 170 kDa transmembrane protein encoded by the multidrug resistance 1 (*mdr1* or *abcb1*) gene (2, 4). It is highly expressed at the apical surface of the intestinal epithelium and plays a critical role in protection of intestinal epithelial cells as well as maintenance of optimal intestinal barrier function (4–6). Loss or dysfunction of P-gp is associated with increased susceptibility to intestinal inflammation, as evidenced both by the link of single nucleotide polymorphisms (SNPs) in *mdr1* and risk of inflammatory bowel disease (IBD) (7–9) and observed development of spontaneous colitis in P-gp knockout (*mdr1a*^−/−^) mice (6, 10, 11). Though multidrug resistance protein 2 (MRP2, *abcc2*) and breast cancer resistance protein (BCRP, *abcg2*) are also expressed at the apical surface of the intestinal epithelium, P-gp is the only related transporter important in maintaining intestinal epithelial barrier integrity (12, 13).

The regulation of P-gp expression and stability is complex and involves numerous transcription factors and post-translational modifications. Transcriptions factors downstream of Wnt signaling, transforming growth factor beta (TGFβ) signaling, and mitogen-activated protein kinase (MAPK) signaling have been shown to increase or decrease P-gp transcription in response to stressors such as heat shock and oxidative stress (14–17), or in development of multi-drug resistance (18, 19). In addition to transcription, functional P-gp expression at the cell membrane requires post-translational modifications including phosphorylation (20). Kinases including PIM-1, protein kinase A (PKA) and protein kinase C (PKC) phosphorylate P-gp and increase its stability (21, 22). One or more of these pathways may be triggered upon exposure to a stressor, toxin or, more recently discovered, bacterial products to converge on P-gp induction (23).

We have previously shown that *Clostridia* and *Bacilli* members of the commensal bacteria of the intestine promote P-gp expression. These bacteria produce short-chain fatty acids (SCFAs) and secondary bile acids, both of which are microbiome-dependent and shown to be required for maximal induction of P-gp expression and function (23). One of the most abundant SCFAs, butyrate, increases P-gp expression in colonic epithelial cell lines (23, 24). Butyrate is a pleiotropic molecule with many roles in the intestine including differentiation of colonocytes, promotion of epithelial barrier integrity, as well as acting as an energy source via beta oxidation in the mitochondria (25–28). Butyrate is involved in many cellular pathways including G protein coupled receptor (GPCR) activation, induction of reactive oxygen species through its utilization in the mitochondria, and activation of nuclear receptors such as nuclear factor erythroid 2-related factor 2 (NRF2) (29–33). Butyrate also inhibits histone deacetylases (HDAC) leading to epigenetic changes and altered gene expression (34–36). Though butyrate has previously been tested alongside other HDAC inhibitors in regulating P-gp, and predicted to increase P-gp transcription via HDAC inhibition (37), this mechanism has not yet been confirmed. Moreover, the roles of GPCR and nuclear receptor activation in P-gp induction have not been determined.

In addition, secondary bile acids are produced by intestinal bacteria including *Clostridia* and *Bacilli* members via deconjugation and conversion of primary bile acids that are secreted by the liver upon ingestion of a meal (38, 39). Three of the more abundant secondary bile acids include lithocholic acid (LCA), deoxycholic acid (DCA), and ursodeoxycholic acid (UDCA). LCA and UDCA have been shown to have anti-inflammatory properties in protection from colitis in mouse models (40, 41). Moreover, these metabolites play a role in protection of the host from infection by pathogens such as Clostridioides difficile (42). LCA, DCA, and UDCA are suggested to interact with nuclear receptors including pregnane X receptor (PXR) and vitamin D receptor (VDR), both of which have been linked to protection from colitis (43–46). VDR has been shown to be involved in LCA induction of P-gp (47). Though LCA and DCA specifically have been shown to activate PXR activity by studying transcriptional reporters in liver cells, as well as linked to P-gp transcription *in vitro* and *in vivo*, the requirement for PXR in secondary bile acid induction of P-gp has not been fully elucidated (41, 48).

We have previously observed that butyrate, LCA, DCA, and UDCA potentiate induction of P-gp protein expression beyond the additive effect of each metabolite alone, reflecting the importance of a cooperative microbial community in producing a combined functional output that communicates to the host. Yet, the mechanism of this combinatorial effect on P-gp induction is unknown. Herein, we describe a new signaling reactome (i.e., regulatory networks and transcription factors) that is uniquely activated in response to the combination of metabolites compared to each metabolite class alone. These findings provide unprecedented insight into a multi-tiered network driven by microbiome-dependent metabolites working in concert to maintain P-gp expression in the intestine in the context of the fluctuating commensal microbiome, to sustain a homeostatic tone in the absence of infection or insult.

## RESULTS

### Butyrate and secondary bile acids together induce a unique transcriptional profile.

Our prior studies identified a “functional core” microbiome of the intestinal gut community, specifically genera within the *Clostridia* and *Bacilli* classes, that is necessary and sufficient for P-gp induction in the intestinal epithelium in mouse models (23). Metagenomic analysis of this core microbial community revealed that abundance of genes involved in short-chain fatty acid and secondary bile acid synthesis, including pyruvate oxidase, choloylglycine hydrolase, and butyryl-CoA dehydrogenase, positively correlated with colonic P-gp expression (23). Intestinal production of butyrate, LCA, DCA, and UDCA is dependent on activity and metabolism by multiple bacterial members of the intestinal microbiota, representing a community functional output. We have previously shown a combination of butyrate, LCA, DCA, and UDCA at physiological concentrations (27, 28, 48–51) potentiates induction of functional P-gp protein in IECs with the metabolite combination inducing higher P-gp expression than either metabolite alone (23). However, given the cellular mechanism of this induction within IECs remains unclear, we sought to determine the intracellular signaling pathways activated by the metabolite combination that contribute to this concerted effect.

**FIG 1:**
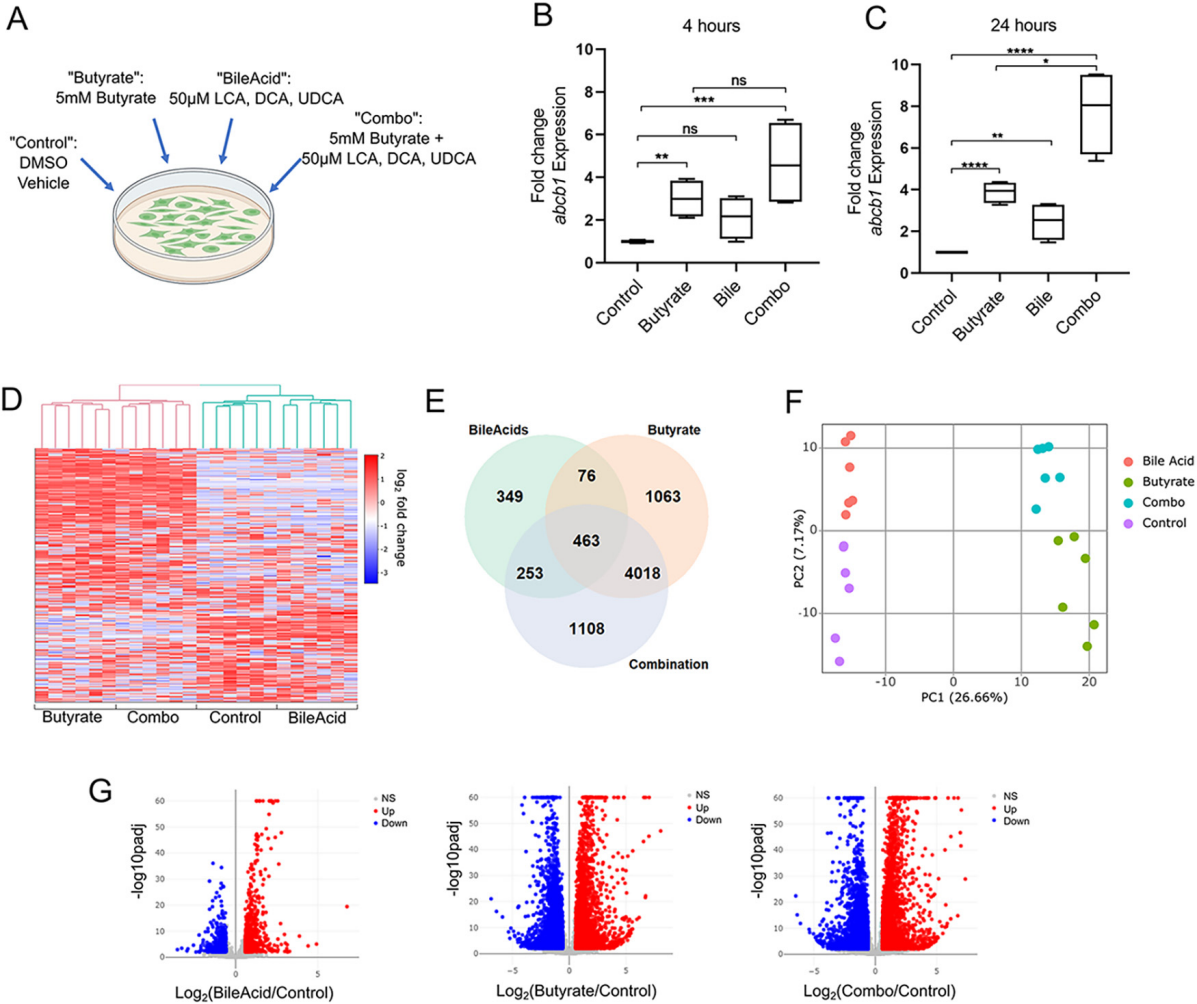
Butyrate and secondary bile acids together induce a unique transcriptional profile. (A) Diagram of treatment of T84 cells with butyrate, LCA, DCA, and/or UDCA, made with BioRender. (B) and (C) T84 cells were incubated with butyrate, LCA, DCA, and/or UDCA, as described in (A) for 4 h (B) or 24 h (C) prior to RNA collection and qPCR analysis for *abcb1* expression. Pooled data from two independent experiments; ns *P* > 0.05; *, *P* < 0.05; **, *P* < 0.01; ***, *P* < 0.001; ****, *P* < 0.0001 one-way ANOVA with Tukey’s multiple-comparison test. (D-G) RNAseq analysis of T84 cells incubated with butyrate, LCA, DCA, and/or UDCA as described in (A) for 4 h. (D) Heat map showing relative expression of top 500 genes with genes in rows and six biological replicates per group shown in columns. (E) Venn diagram showing number of genes differentially expressed in cells after each treatment (cut-off *P* < 0.01, fold change >1.5). (F) Principal-component analysis of top 500 differentially expressed genes. Points represent individual samples, colors represent treatment as indicated in the legend. (G) Volcano plot of log_2_(fold change) of each treatment versus control determined by DESEQ2 analysis, cut-off set to *P* < 0.01, fold change >1.5.

Since P-gp expression can be regulated both transcriptionally and post-translationally, we first looked at the mRNA level of P-gp after incubation with these metabolites and found that at an early time point of 4 h, *ABCB1* mRNA is not significantly increased in the metabolite combination compared to butyrate or bile acids alone (Fig. 1A and B). However, at 24 h, there is a synergistic induction of *ABCB1* mRNA in the presence of both classes of metabolites (butyrate and secondary bile acids) (Fig. 1A and C). This suggests a transcriptional program converging to sustain P-gp induction that is uniquely activated by the combination of metabolites compared to either metabolite alone. P-gp regulation by these metabolites could include a unique transcriptional program that increases expression of factors such as transcription factors and kinases including Pim1, PKA, and PKC which, once translated, contribute at a later time point to further increase P-gp transcription as well as increase stability of newly translated P-gp through phosphorylation. To determine the cellular networks being activated by these metabolites, we performed RNA sequencing (RNAseq) on T84 cells subjected to butyrate alone, a combination of the three secondary bile acids (LCA, DCA, and UDCA), a combination of all four metabolites (“combo”) or vehicle control, for 4 h, as shown in Fig. 1A RNAseq analysis revealed considerable changes to the transcriptome with butyrate treatment in support of previous studies (52, 53) (Fig. 1D, E and G). While the effect of bile acids is more limited (Fig. 1D, E and G), bile acid-treated cells clustered distinctly from DMSO control (Fig. 1F) and exhibited a unique transcription profile of 349 differentially expressed genes compared to DMSO control (Fig. 1E). Strikingly, even with global changes due to butyrate, we observed a set of 1108 genes uniquely differentially expressed by the combination of metabolites that are distinct from each metabolite class alone (Fig. 1E). We therefore pursued unique pathways activated by the metabolite combination that converge to potentiate induction of P-gp, as well as resolve the pathways by which P-gp is induced downstream of butyrate alone and bile acids alone.

### Metabolite combination activates several unique pathways linked to P-gp upregulation.

**FIG 2:**
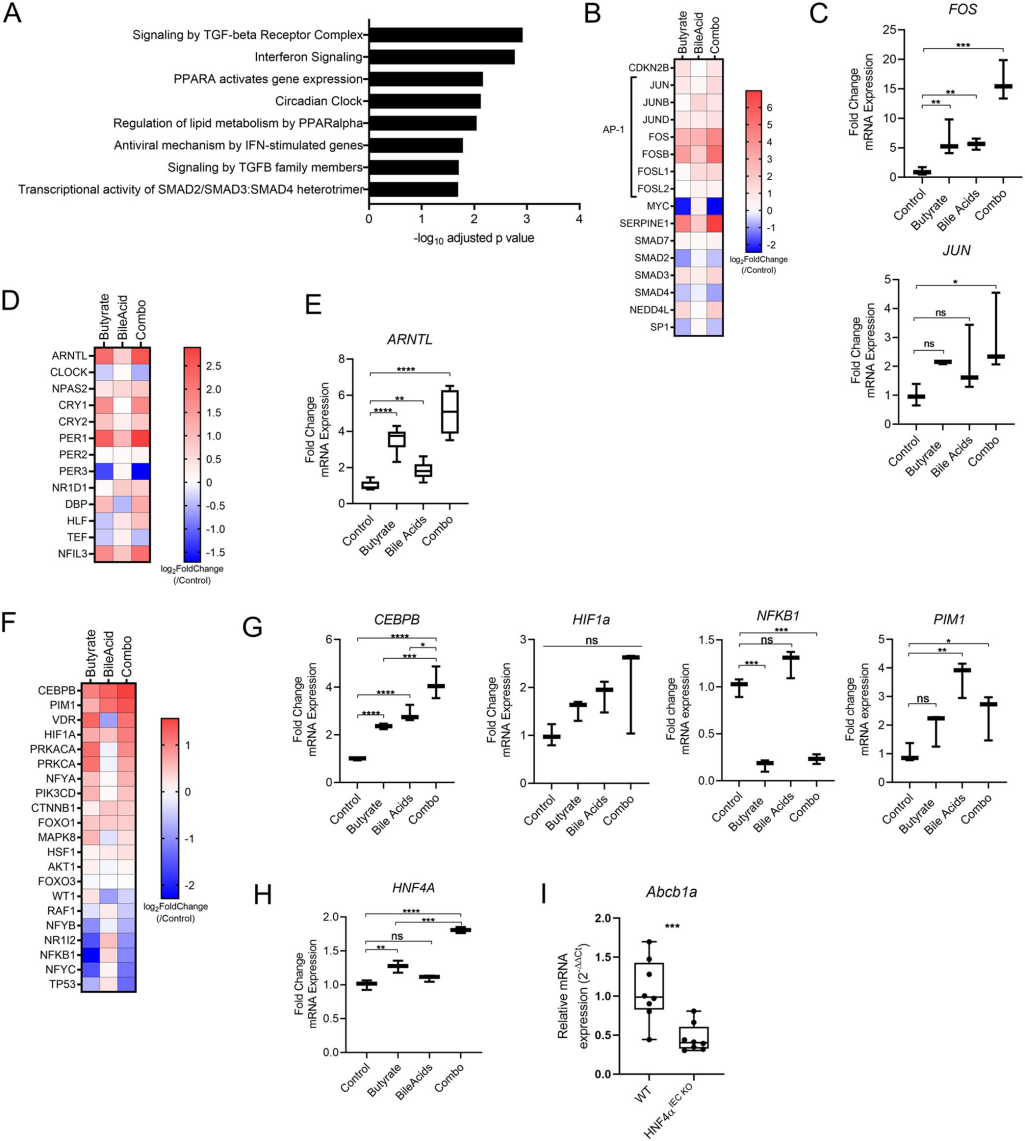
Metabolite combination activates multiple pathways related to P-gp induction. (A) Pathways (Reactome) significantly and uniquely enriched in metabolite combination-treated T84 cells compared to butyrate- or bile acid-treated T84 cells, determined by g:Profiler analysis of RNAseq data (Fig. 1). (B) Heat map of RNAseq data showing relative expression of genes related to TGFβ signaling. (C) qPCR analysis of *FOS* and *JUN* expression in T84 cells treated with butyrate, bile acids or a combination for 4 h compared to DMSO control as in Fig. 1B (D) Heat map of RNAseq data showing relative expression of genes related to circadian clock signaling. (E) qPCR analysis of *ARNTL* expression in T84 cells as in (C). (F) Heat map of RNAseq data showing relative expression of genes related to P-gp regulation. (G) qPCR analysis of *CEBPB*, *PIM1*, *HIF1a*, and *NFKB1* expression in T84 cells as in (C). (H) qPCR analysis of *HNF4A* expression in T84 cells as in (C). (C, E, G, H) Data are pooled from 3–6 biological replicates. ns *P* > 0.05; *, *P* < 0.05; **, *P* < 0.01; ***, *P* < 0.001; ****, *P* < 0.0001 by one-way ANOVA with Tukey’s multiple-comparison test. (I) qPCR analysis of *Abcb1a* expression in colon tissue of wild-type (WT) and intestinal epithelial knockout of HNF4α (HNF4α^IEC KO^). N = 8 per genotype, N = 4 female and N = 4 male mice. ***, *P* < 0.001 by unpaired *t test*.

We initially determined the mechanism by which the combination of butyrate and bile acids potentiate induction of P-gp, reflecting communication to the host by a complex microbiome community to effect a maximal response. Given the multiple layers of P-gp regulation at transcription and post-translation stages, we hypothesize this mechanism includes regulation of multiple factors that contribute temporally at these different stages, including transcription factors, transcription co-factors, and kinases. Therefore, we first focused on pathways uniquely activated in cells treated with the combination of butyrate and bile acids but not by either metabolite class alone (Fig. 2A). Among these, three sets of pathways related to transforming growth factor β (TGFβ) signaling and SMAD2/SMAD3:SMAD4 transcription factors are significantly enriched in butyrate and bile acid combination-treated cells (Fig. 2A to C). TGFβ signaling leads to transcription factor oligomerization in the nucleus and subsequent transcription (54). Complexes of SMAD proteins interact with other transcription factors including AP-1 family members (54). TGFβ signaling and the AP-1 transcription factor complex are linked to both basal constitutive expression of P-gp as well as induced expression in response to stress stimuli (16, 18). The AP-1 complex is composed of transcription factors including Jun-Fos family members, two of which, c-Jun (*JUN*) and c-Fos (*FOS*), are linked to increased P-gp promoter activity (16, 18), particularly in induction driven by incubation of IECs with supernatants from *Lactobacilli* cultures (55). Expression of c-Jun and c-Fos are both significantly increased in cells treated with both metabolite classes (Fig. 2C), suggesting they may contribute to furthered increase in P-gp transcription.

An additional pathway enriched in the metabolite combination is related to the circadian clock (Fig. 2A, D, and E), including regulation of the main clock regulators, *ARNTL* and *CLOCK*, as well as many other circadian clock-related genes (Fig. 2D and E). Circadian clock and diurnal oscillations have been observed in the microbiota composition itself as well as in microbiota-driven phenotypes in the intestine and liver (56–58). Furthermore, circadian clock and diurnal oscillations have also been observed in expression of P-gp (57, 59). Since we observed a dependence of mouse intestinal P-gp expression on the resident microbiota (23), we next determined whether the microbiota contributes to the oscillatory pattern of intestinal P-gp expression. While diurnal oscillation of P-gp mRNA expression was observed that aligns with previous findings (57, 60), treatment of mice with the broad-spectrum antibiotic cefoperazone (CFP), previously shown to significantly reduce the intestinal bacterial load and P-gp expression (23), reduced the intensity of P-gp expression but did not affect the pattern or amplitude of P-gp oscillation (Fig. S1A and B). Additionally, we saw no change to the expression of the clock regulator gene, *ARNTL*, in antibiotic-treated mice (Fig. S1A). We conclude the circadian clock genes and microbiota are not linked in promoting P-gp expression, rather the microbiota provides an additional exogenous input for increased P-gp expression above constitutive basal levels.

10.1128/mbio.01993-22.1 FIG S1 Circadian clock genes not involved in microbiome regulation of P-gp. (A) Wild-type mice were treated with cefoperazone (CFP) for 7 days prior to euthanasia of a group (N = 3) every 4 h for a 24-hour light-dark cycle. X-axes represent Zeitgeber time (ZT) in hours with ZT0 indicating the onset of the light cycle. Shown are relative gene expression of *Abcb1a* and *ARNTL* at each timepoint. (B) Amplitude (peak/trough) of *Abcb1a* expression in untreated and CPF-treated mice. *P* > 0.05 by unpaired *t*-test. Download FIG S1, TIF file, 0.1 MB. Copyright © 2022 Foley et al. 2022 Foley et al. https://creativecommons.org/licenses/by/4.0/ This content is distributed under the terms of the Creative Commons Attribution 4.0 International license.

To additionally interrogate signaling pathways that may be activated by the metabolite combination, we next probed our RNAseq data set for all factors previously linked to P-gp expression (16), including those involved in early transcription events as well as late post-translational modifications. We found that expression of several of these factors were increased or decreased by the metabolite incubation (Fig. 2F). *CEBPB* and *HIF1A* are transcription factors that respond to inflammation and oxidative stress, respectively, and have been shown to increase P-gp transcription (61–64). *CEBPB* is significantly increased by the metabolite combination and may likely play a role in furthering P-gp induction (Fig. 2F and G). While our results show variability in *HIF1A* induction, a low level of induction of this transcription factor may contribute to furthered P-gp induction (Fig. 2F and G).

NF-κB has been shown to increase or decrease transcription of P-gp in a cell-type specific manner; in non-multidrug resistant cells, NF-κB suppresses P-gp transcription through interaction with an inhibitory transcription factor complex that binds the P-gp promoter region (65). mRNA expression of *NFKB1* is significantly decreased with butyrate and combination treatment of IECs (Fig. 2F and G), and this reduction may also play a role in P-gp induction. In addition to transcriptional regulation, P-gp also undergoes extensive post-translational modifications including glycosylation, trafficking, and phosphorylation. Pim-1 kinase has been shown to phosphorylate P-gp to increase its stability and surface localization. Pim-1 kinase mRNA expression is significantly increased with the metabolite combination as well as bile acids alone and may play a role in increasing stability of newly translated P-gp protein (Fig. 2F and G).

Lastly, hepatocyte nuclear factor 4α (HNF4α) is a transcription factor not yet closely linked to P-gp in the intestine. Knockdown of HNF4α in hepatocytes is associated with reduced *ABCB1* mRNA expression (66), but the mechanism is unclear. HNF4α is also expressed in IECs, is reduced in ulcerative colitis patients, and IEC-specific deletion of HNF4α in mice leads to spontaneous colitis that closely mirrors the association of P-gp in protection from colitis (67). Remarkably, the combination of metabolites significantly increased HNF4α mRNA expression more than either metabolite individually (Fig. 2H). Moreover, in colon tissue isolated from untreated mice with IEC-specific deletion of HNF4α (HNF4α^ΔIEC^), we observed a significant reduction of *Abcb1a* mRNA expression compared to wild-type mice (Fig. 2I), supporting HNF4α plays a role in P-gp transcription in IECs. Though not significantly reduced, P-gp protein expression in IECs from HNF4α^ΔIEC^ mice is dysregulated with increased variability (Fig. S2), further pointing to a requirement for HNF4α for stable expression of P-gp. Altogether these data unveil multiple intracellular signaling networks that are uniquely activated by the combination of butyrate and secondary bile acids that involve both known and previously unknown regulators of P-gp expression.

10.1128/mbio.01993-22.2 FIG S2 P-gp protein expression dysregulated in HNF4α^ΔIEC^ mouse intestinal epithelium. Western blot of P-gp protein expression in wild-type and HNF4α^ΔIEC^ mice, each lane representing a replicate mouse within each group. N = 5 female mice per group. Densitometry data using GAPDH as internal loading control, *P* > 0.05 by unpaired *t*-test. Download FIG S2, TIF file, 0.2 MB. Copyright © 2022 Foley et al. 2022 Foley et al. https://creativecommons.org/licenses/by/4.0/ This content is distributed under the terms of the Creative Commons Attribution 4.0 International license.

### Butyrate induces P-gp transcription via HDAC inhibition but not NRF2.

**FIG 3:**
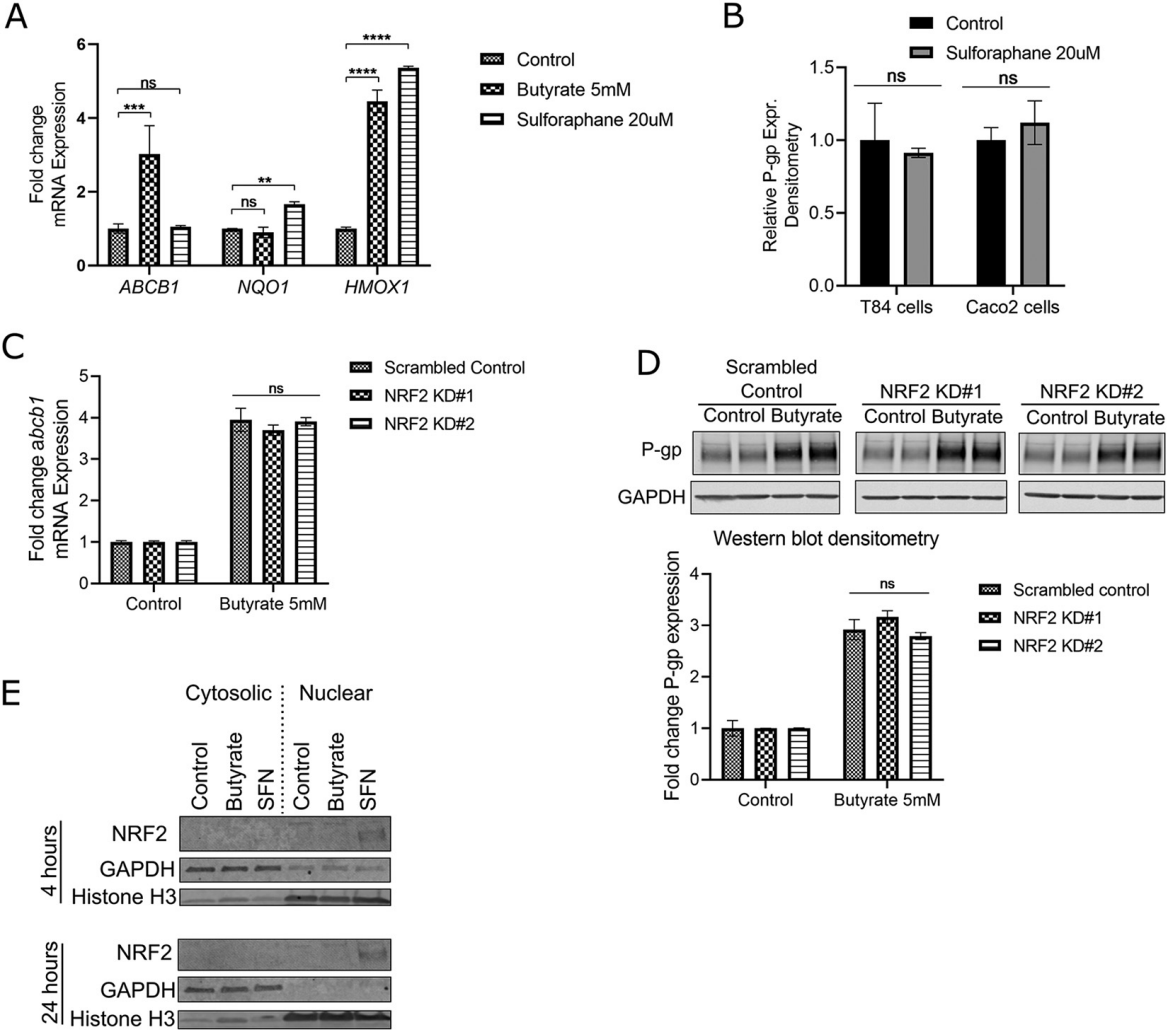
Butyrate induces a transcriptional program to increase P-gp expression independent of NRF2. (A) Fold change expression of *ABCB1*, *NQO1*, and *HMOX1* in T84 cells treated with butyrate or sulforaphane for 4 h. One-way ANOVA with Dunnett’s multiple-comparison test, ns *P* > 0.05; **, *P* < 0.01; ***, *P* < 0.001; ****, *P* < 0.0001. (B) Densitometry analysis of P-gp protein expression by Western blot in T84 cells and Caco2 cells after 24 h incubation with sulforaphane. ns *P* > 0.05 by unpaired *t test* for each cell line. (C) Fold change expression of *ABCB1* in T84 scrambled control and NRF2 knockdown cells after 4 h incubation with butyrate. ns *P* > 0.05 by one-way ANOVA. (D) Representative Western blot and densitometry for P-gp expression in T84 scrambled control and NRF2 knockdown cells after 24 h incubation with butyrate. ns *P* > 0.05 by one-way ANOVA. (E) Representative Western blot of NRF2 nuclear localization in T84 cells after incubation with butyrate (20 mM) or sulforaphane (20 μM) for 4 h and 24 h. GAPDH and Histone H3 detected as controls for cytoplasmic and nuclear lysate fractions, respectively. (A-E) All data representative of at least two independent experiments.

**FIG 4:**
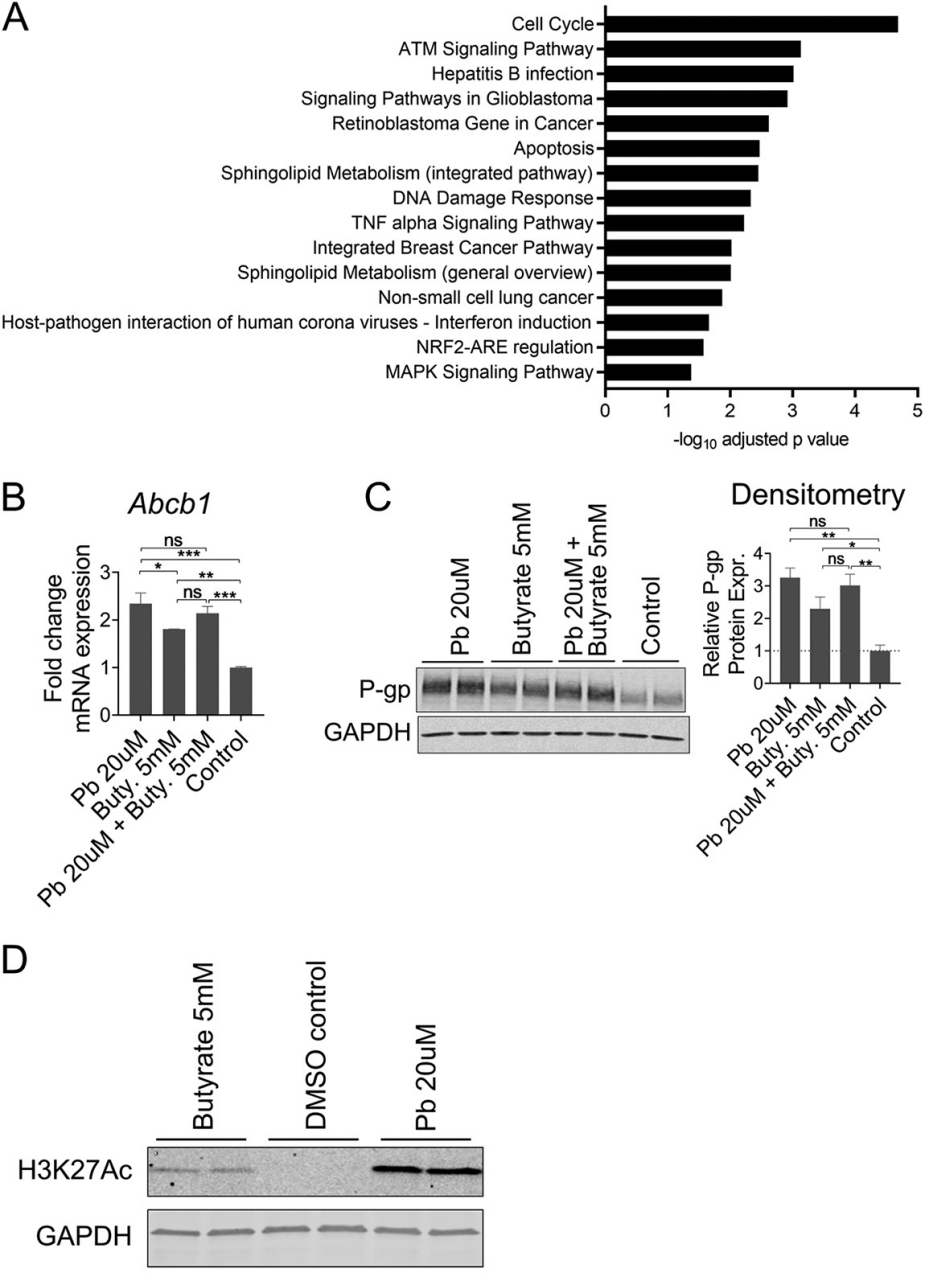
Butyrate increases P-gp expression via HDAC inhibition. (A) Significantly enriched pathways (wikipathways) in butyrate-treated T84 cells determined by g:Profiler analysis of RNAseq data (Fig. 1). (B) Fold change expression of *Abcb1* by qPCR of T84 cells after 4 h incubation with panobinostat (Pb, 20 μM), butyrate (Buty., 5 mM), or a combination compared to DMSO vehicle (control). Ns *P* > 0.05; **, *P* < 0.01; ***, *P* < 0.005; by one-way ANOVA with Tukey’s multiple-comparison test. (C) Representative Western blot and densitometry of P-gp expression in T84 cells after 24 h incubation with panobinostat or butyrate as in Fig. 3B, ns *P* > 0.05; *, *P* < 0.05; **, *P* < 0.01 by one-way ANOVA with Tukey’s multiple-comparison test. (D) Western blot of histone 3 lysine 27 acetylation mark (H3K27Ac) in T84 cells after 24 h incubation with panobinostat or butyrate as in Fig. 4B, with GAPDH as loading control. (B-D) Representative data of at least two independent experiments.

We next sought to determine the pathways by which P-gp is induced downstream of butyrate alone and secondary bile acids alone. Butyrate can activate cellular signaling through multiple pathways and receptors (29–33). To begin determining the pathway by which butyrate induces P-gp in IECs, we first looked at involvement of the transcription factor NRF2, previously linked to P-gp regulation. NRF2 binds to gene promoter regions containing an antioxidant response element (ARE) to coordinate antioxidant and detoxification responses, termed the NRF2-ARE pathway (68). Prior studies have described a link between the transcriptional activity of NRF2 and *ABCB1* mRNA induction, as well as butyrate induction of NRF2 activation and translocation from the cytoplasm to the nucleus (69, 70). Though our RNAseq data set shows mixed regulation of NRF2 target genes in butyrate-treated cells compared to vehicle control (Fig. S3A), we assessed whether NRF2 was involved in butyrate upregulation of P-gp. We found that sulforaphane (SFN), an inducer of oxidative stress that leads to NRF2 activation (69), did increase mRNA expression of two main NRF2 target genes, *NQO1* and *HMOX1* (Fig. 3A). However, SFN treatment did not change P-gp expression at the mRNA or protein levels in T84 cells or Caco2 cells (Fig. 3A and B, and Fig. S3B). Additionally, while butyrate increases mRNA expression of *ABCB1* as well as one, but not both, NRF2 target genes, short hairpin RNA (shRNA) knockdown of NRF2 in T84 cells, resulting in reduced NRF2 mRNA and protein expression (Fig. S3C and S3D), did not affect baseline or butyrate induction of P-gp at the mRNA or protein levels (Fig. 3C and D). Moreover, we did not observe nuclear localization of NRF2 after incubation with butyrate as was apparent with SFN (Fig. 3E and Fig. S2E). Though pathway enrichment analysis of the RNAseq data set by gProfiler analysis revealed NRF2-ARE regulation as being significantly enriched by butyrate treatment of T84 cells (Fig. 4A), our results oppose published findings by determining NRF2 is not required for butyrate induction of P-gp.

10.1128/mbio.01993-22.3 FIG S3 NRF2 is not required for butyrate induction of P-gp. (A) Heat map of RNAseq data showing relative expression of genes related to NRF2 signaling in T84 cells incubated with butyrate as described in Fig. 1. (B) Representative western blot of P-gp expression after incubation of T84 cells and Caco2 cells with butyrate or sulforaphane (SFN) compared to DMSO vehicle control for 24 h. Densitometry in Fig. 3B. (C) qPCR analysis of *NFE2L2* in T84 scrambled control and NRF2 shRNA-expressing cells to validate knockdown. ***, *P* < 0.001 by one-way ANOVA with Dunnett’s multiple comparisons test. (D) Relative western blot and densitometry of NRF2 expression in T84 scrambled control and NRF2 shRNA-expressing cells to validate knockdown. T84 cells were incubated with proteasome inhibitor MG132 or sulforaphane (SFN) for 6 h to increase NRF2 protein to detectable levels. *, *P* < 0.05; **, *P* < 0.01 by Dunnett’s multiple comparisons test. (E) Representative western blot of NRF2 nuclear localization in Caco2 cells after incubation with butyrate (20mM) or sulforaphane (20μM) compared to DMSO vehicle control for 4 h and 24 h. GAPDH and Histone H3 detected as controls for cytoplasmic and nuclear lysate fractions, respectively. Download FIG S3, TIF file, 0.5 MB. Copyright © 2022 Foley et al. 2022 Foley et al. https://creativecommons.org/licenses/by/4.0/ This content is distributed under the terms of the Creative Commons Attribution 4.0 International license.

Pathway enrichment analysis of the RNAseq data set more prominently revealed regulation of MAPK signaling and pathways related to cell cycle and carcinogenesis enriched in butyrate-treated T84 cells compared to vehicle control (Fig. 4A). MAPK signaling is involved in many pathways and found downstream of GPCR signaling. Butyrate has been shown to activate signaling cascades leading to transcription through GPCRs including GPR41, GPR43, and GPR109A, all of which are classified as G_αi_-associated GPCRs (29–31). We therefore tested whether G protein inhibitors could block induction of P-gp by butyrate using Pertussis toxin (PTx), an inhibitor of G_αi_ subunits, and BIM-46187, a reported inhibitor of all G_α_ subunits (71, 72), as well as inhibitors of G_βγ_ subunit (Gallein) and β-arrestin (Barbadin) as controls (71, 73). Butyrate induction of P-gp protein was not affected by the G protein inhibitors tested, suggesting a GPCR-independent mechanism (Fig. S4).

10.1128/mbio.01993-22.4 FIG S4 Butyrate does not upregulate P-gp via GPCR activity. T84 cells were pre-incubated with G protein inhibitors barbadin, BIM-46187, Gallein, or Pertussis Toxin (PTx) prior to incubation with butyrate and G protein inhibitors for 24 h. (A) Representative western blots of P-gp expression in T84 cells. (B) Densitometry of western blots in (A), represented as fold induction of P-gp over baseline with G protein inhibitor in the absence of butyrate. *P* > 0.05 by one-way ANOVA for experiment 1 (Exp 1) and *P* > 0.05 by unpaired *t*-test for experiment 2 (Exp 2). Download FIG S4, TIF file, 0.2 MB. Copyright © 2022 Foley et al. 2022 Foley et al. https://creativecommons.org/licenses/by/4.0/ This content is distributed under the terms of the Creative Commons Attribution 4.0 International license.

Butyrate is also reported to inhibit HDACs, leading to increased histone acetylation. Cell cycle and carcinogenesis pathways, found to be enriched in butyrate-treated cells compared to vehicle control, are often associated with chromatin modifications (74). We therefore tested whether butyrate induces P-gp through HDAC inhibition. Butyrate has been previously shown to induce P-gp and this induction has been correlated to that induced by other HDAC inhibitors (37), however a direct link of butyrate acting through HDAC inhibition to induce P-gp has not yet been shown. We utilized the pan-HDAC inhibitor Panobinostat and showed induction of both *ABCB1* mRNA and P-gp protein with either Panobinostat or butyrate, but no further induction with a combination of the two in support of their having the same mechanism of action (Fig. 4B and C). Additionally, this induction of P-gp by either Panobinostat or butyrate was accompanied by acetylation of the lysine27 residue of histone H3 (H3K27), an acetylation mark shown to be associated with P-gp induction with increasing multi-drug resistance in cancer cells (75) (Fig. 4D). Altogether these data support and confirm that butyrate induces P-gp expression through HDAC inhibition.

### Secondary bile acids activate PXR and VDR upstream of P-gp.

**FIG 5:**
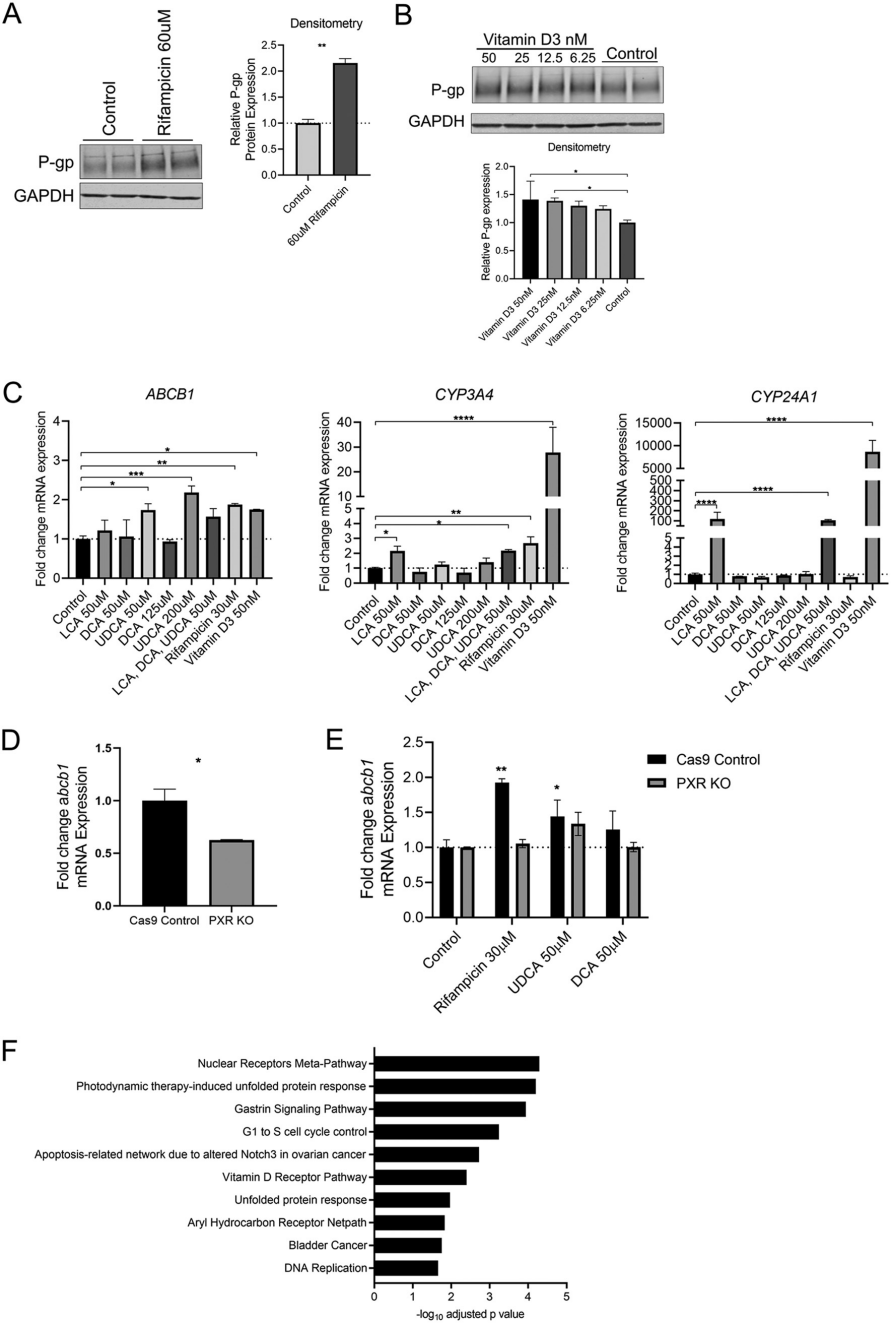
Bile acids activate PXR and VDR nuclear receptors upstream of P-gp induction. (A) Representative Western blot and densitometry of P-gp expression in T84 cells after 24 h incubation with rifampicin versus DMSO control. **, *P* < 0.01 by unpaired *t test*. (B) Representative Western blot and densitometry pooled from two independent experiments of P-gp expression in T84 cells after 24 h incubation with vitamin D3 versus DMSO control. *, *P* < 0.05 by one-way ANOVA with Dunnett’s multiple-comparison test. (C) qPCR analysis of *ABCB1*, *CYP3A4*, and *CYP24A1* expression in T84 cells treated with compounds as shown for 4 h compared to DMSO control. One-way ANOVA with Dunnett’s multiple-comparison test, *, *P* < 0.05; **, *P* < 0.01; ***, *P* < 0.001; ****, *P* < 0.0001. (D) qPCR analysis of *ABCB1* expression at baseline in Cas9 control and PXR KO T84 cells. *, *P* < 0.05 by unpaired *t test*. (E) qPCR analysis of *ABCB1* expression in Cas9 control and PXR KO T84 cells after 4 h incubation with rifampicin or bile acids as indicated. One-way ANOVA with Dunnett’s multiple-comparison test performed for each cell line, *, *P* < 0.05; **, *P* < 0.01 for each condition versus DMSO vehicle control. (A-E) Representative data of at least two independent experiments. (F) Significantly enriched pathways (wikipathways) in butyrate-treated T84 cells determined by g:Profiler analysis of RNAseq data (Fig. 1).

Secondary bile acids are structurally distinct from butyrate and have been shown to activate cellular receptors including nuclear receptors that function as transcription factors or co-factors. *ABCB1* is a known target of both pregnane X receptor (PXR) and vitamin D receptor (VDR). Therefore, we pursued clarifying the involvement of these two receptors in secondary bile acid induction of P-gp. Rifampicin is an agonist of PXR, while Vitamin D_3_ (calcitriol) is an agonist of VDR, and both have been shown to increase P-gp expression in published studies (41, 47, 48), however the role of PXR and/or VDR in the induction of P-gp in response to LCA, DCA, and UDCA has not been determined. We have confirmed and expanded on previous work to show Rifampicin and Vitamin D_3_ induce P-gp at both the transcriptional and protein level (Fig. 5A to C). While there is some overlap between PXR and VDR DNA binding sites, LCA is a strong inducer of the VDR target gene *Cyp24A1*, while LCA shows less induction of the PXR target gene *Cyp3A4* (Fig. 5C). Whether this is due to differing baseline expression levels of PXR and VDR is unclear.

LCA has been published to increase P-gp transcription via VDR (47), however the involvement of PXR or VDR in upregulation of P-gp by DCA or UDCA has not been shown. DCA and UDCA activate *Cyp3A4* transcription, but not *Cyp24A1*, supporting DCA and UDCA activation of PXR but not VDR. To determine whether PXR is required for DCA and/or UDCA induction of P-gp, we performed CRISPR/Cas9 knockout of PXR in T84 cells (Fig. S5). We confirmed loss of rifampicin-induced *Cyp3A4* transcription (Fig. S5) that coincided with a reduction of *Abcb1* transcription at baseline (Fig. 5D), as well as loss of rifampicin induction of *Abcb1* transcription (Fig. 5E). While PXR knockout had no effect on UDCA induction of P-gp, the effect of DCA was eliminated (Fig. 5E). These data suggest PXR is required for DCA induction of P-gp, but not required for UDCA induction (Fig. 5E). These data also highlight the complex involvement of multiple factors in secondary bile acid-induction of P-gp expression.

10.1128/mbio.01993-22.5 FIG S5 CRISPR/Cas9 knockout of PXR function. qPCR analysis of *CYP3A4* in Cas9 control T84 cells and PXR KO T84 cells after 4 h incubation with 30μM rifampicin versus DMSO vehicle control. *, *P* < 0.05, ns *P* > 0.05, unpaired *t*-test. Representative data of at least two independent experiments. Download FIG S5, TIF file, 0.08 MB. Copyright © 2022 Foley et al. 2022 Foley et al. https://creativecommons.org/licenses/by/4.0/ This content is distributed under the terms of the Creative Commons Attribution 4.0 International license.

Pathway enrichment analysis using gProfiler of the RNAseq data set of bile acid-treated cells versus vehicle control revealed nuclear receptors meta-pathway and the vitamin D receptor pathway among those significantly enriched (Fig. 5F), in support of our findings (Fig. 5A to E). Further, this analysis also highlights additional pathways that may be involved, including apoptosis pathways and aryl hydrocarbon receptor activity (Fig. 5F).

## DISCUSSION

P-gp expression in the intestinal epithelium is critical for maintenance of homeostasis and suppression of aberrant neutrophil infiltration that is characteristic of ulcerative colitis. Yet the mechanisms underlying its relatively high expression in the colon compared to other healthy tissues is still being unraveled. Previous work has shown the healthy microbiome is responsible for high induction of P-gp above baseline. This work has identified key components of the signaling networks shown to be directly involved in regulation of P-gp expression in IECs by either butyrate or the secondary bile acids LCA, DCA, and UDCA. While butyrate is a known HDAC inhibitor and expression of P-gp, like many other proteins, is likely increased by histone modifications, for the first time we have shown that P-gp induction in IECs by butyrate is directly linked to the HDAC inhibition activity of butyrate.

Bile acids overall are suggested, and in some cases shown, to be agonists of the nuclear receptors PXR and VDR. However, we uniquely discerned the involvement of PXR in P-gp induction by LCA, DCA, and UDCA. LCA and UDCA have been shown to protect from colitis (40, 41). Likewise, activation of PXR protects from induced colitis in mouse models (44). The expression profile of PXR and its target gene Cyp3A4 mirror P-gp at baseline, and both are decreased in mouse models of colitis and human ulcerative colitis similar to P-gp (69, 76). It has been previously suggested that Cyp3A4 and P-gp play synchronous roles in detoxification responses to promote cell and tissue health (77). Our findings highlight another example in which the two are co-regulated. VDR has been previously linked to P-gp induction in response to LCA (47). While DCA and UDCA have been suggested as potential agonists of VDR as well (43), our findings show limited induction of VDR activity by DCA and UDCA, and instead that DCA increases expression of P-gp through PXR activity. Beyond confirmation of these suggested links based on previous studies, our RNAseq analysis has highlighted additional pathways that may also be involved in secondary bile acid regulation of P-gp.

**FIG 6:**
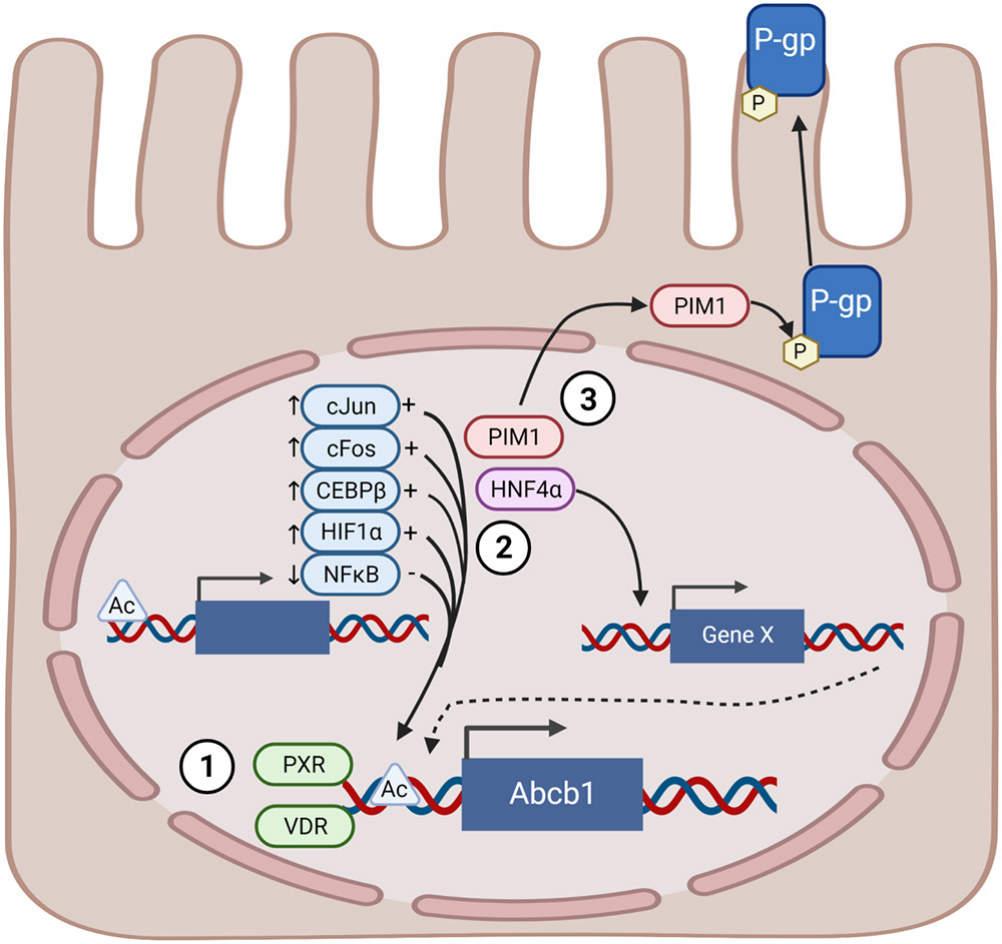
Multi-tiered model of P-gp regulation by butyrate and bile acids. Tier 1: Butyrate and bile acids increase P-gp transcription via HDAC inhibition and nuclear receptor activation (PXR and VDR), respectively. Tier 2: The combination of butyrate and bile acids increases expression of transcription factors that activate, and decrease expression of transcription factors that repress, P-gp transcription resulting in increased P-gp expression. Tier 3: These metabolites increase expression of factors including Pim-1 kinase that contribute to post-translational modifications of P-gp. Diagram created with BioRender.

More notable, our work has unveiled networks of intracellular signaling that are significantly triggered in IECs only when the two classes of metabolites are combined, and these networks converge to intensify the expression of P-gp. We have highlighted key signaling pathways that have already been linked to P-gp induction and likely play a role in response to butyrate and secondary bile acids. Based on our findings and supporting literature, we propose a multi-faceted model: confirming our hypothesis, the first tier of the model for the synergistic effect is through overall increased access of chromatin to transcription factors and other co-factors in the presence of butyrate; butyrate increases acetylation, and therefore accessibility, of chromatin, which may increase access of factors downstream of bile acids that would be otherwise nonfunctional, increasing overall P-gp transcription (Fig. 6).

We also uncovered a second tier of promoting P-gp expression related to the kinetics of induction of both P-gp and its regulators (Fig. 1 and 6). Expression of transcription factors linked to P-gp, including cJun, cFos, CEBPβ, and HIF1α, is increased by the metabolite combination at an early time point. We posit that as protein levels of these transcription factors increase, this will further shift the cell to more transcription of P-gp and their other targets at later time points. We have also identified expression of HNF4α is significantly increased in the presence of butyrate and bile acids, and that P-gp expression in IECs is partially dependent on HNF4α as seen in the IEC conditional HNF4α knockout mice (Fig. 2I). It has been shown that HNF4α interacts with the P-gp promoter, though its binding decreases in germ-free mice upon colonization with a specific pathogen-free microbiota (78), where we have shown P-gp expression is increased (23). Therefore, HNF4α may promote P-gp expression indirectly through interaction with yet other promoters or co-factors. The third tier underlying butyrate and secondary bile acid synergistic induction of P-gp expression is through post-translational modifications such as phosphorylation by Pim-1 (Fig. 6). In this way, the inputs from the microbial community work in concert to provide critical induction and maintenance of P-gp expression.

The multiple layers of signaling networks that contribute to regulation of P-gp have to date been underappreciated. P-gp is an evolutionarily conserved transporter and the host has evolved many pathways to maintain its expression in the intestinal epithelium due to its diverse and important roles in maintaining intestinal health. The intestinal microbiome contributes to maintenance of intestinal health, yet has been shown to fluctuate between various healthy states, often driven by environmental factors such as diet (58, 79–81). The intestinal microbiome has evolved with the host, thus we hypothesize the activation of multiple signaling networks converging on P-gp transcription by this combination of microbiome-derived metabolites is a mechanism that has evolved between the bacteria and mammalian intestine in the context of the fluctuating commensal microbiome to ensure high expression of P-gp at the intestinal surface.

Much like the functional output of the microbial community is complex, the array of intracellular networks in the IEC triggered by these microbial metabolites is equally complex. This study raises further questions regarding what other networks are being co-regulated with P-gp. We have shown that endocannabinoids released by P-gp suppress aberrant inflammation via inhibition of neutrophil infiltration (1). Studies are beginning to unravel how endocannabinoids may alter the microbiome community (82). Moreover, these findings identify new mechanisms for the roles for PXR and VDR in intestinal health via induction of P-gp in response to the microbiota. Additionally, TGFβ signaling can promote pro-inflammatory and immunosuppressive responses depending on the cytokine context; TGFβ signaling has also shown to be important in anti-microbial peptide production, which are important for maintaining the mucosal barrier as well as shaping the local microbiota. Taken together, these findings suggest that P-gp along with VDR activation, PXR activation, and TGFβ signaling, could constitute an immune-suppressive program that is co-regulated by the microbiota and supports homeostasis in the intestine.

Overall, these findings identify new links of the microbiota and protection from colitis, including novel findings of how multiple classes of bacterial metabolites from the microbiota can synergistically promote an intestinal phenotype that in turn involves many integrated signaling networks. Further, while many of the signaling pathways have been associated with multi-drug resistance in other tissue types, these microbial metabolites are largely limited to the intestine lumen and are primarily taken up by the IECs (butyrate) or recycled to the liver (bile acids) (83, 84). Therefore, utilizing the mechanisms identified here that underlie the beneficial effect of these metabolites and P-gp induction in the local context of the intestine can be regarded as a potential therapeutic opportunity in ulcerative colitis and other inflammatory conditions of the intestine.

## MATERIALS AND METHODS

### Materials.

Sodium butyrate, sodium deoxycholate, ursodeoxycholic acid, doxycycline, MG132, sulforaphane, cefoperazone, and BIM-46187 were purchased from Sigma. Lithocholic acid, Panobinostat, and pertussis toxin were purchased from Cayman Chemical. Gallein was purchased from Tocris Bioscience. Barbadin was purchased from Axon MedChem, LLC.

### Cell lines.

Human intestinal epithelial cell lines (T84, Caco2, and HEK293) were maintained in a humidified incubator (5% CO_2_, 95% air, 37°C). T84 adenocarcinoma colon cells (ATCC) were maintained in growth media (DMEM:Ham’s F12 media, 7.5% FBS (HyClone) and 100U/mL penicillin/streptomycin (Gibco) at passages 60–70). For assays, cells were seeded onto collagen-coated tissue culture plates and allowed to reach confluence before incubation with compounds.

Caco2 cells (a gift from Dr. Pradeep Dudeja) were maintained in growth media (EMEM) (ATCC), 10% FBS (Optima) and 100U/mL penicillin/streptomycin (Gibco) at passages 5–20. For assays, cells were seeded onto tissue culture plates or collagen-coated 0.4 μm Transwell (Corning) plates and allowed to reach confluence. Cells seeded on plastic were used for experiments 1 week after reaching confluence. Cells seeded on Transwells were differentiated for 3 weeks post-seeding.

HEK293 cells (a gift from William McDougall) were maintained in DMEM growth media with 10% FBS and 100U/mL penicillin/streptomycin.

### RNA isolation from human cell lines.

Cells were washed with phosphate-buffered saline (PBS) and either stored suspended in Cell Protect Reagent (Qiagen) at −20°C or directly lysed in Buffer RLT (Qiagen). Total RNA was extracted using the RNeasy Mini kit (Qiagen), with a DNA removal step using RNase-free DNase I (Qiagen). Purity and concentration were measured by NanoDrop.

### RNA sequencing.

RNA was isolated as described above from T84 cells incubated with butyrate (5 mM), LCA/DCA/UDCA (50 μM each), a combination (5 mM butyrate, 50 μM each LCA, DCA, UDCA) or DMSO vehicle for 4 h. RNA samples were isolated from six sets of biological replicates seeded from separate cell passages. RNA integrity was analyzed using a 5300 Fragment Analyzer (Agilent) and concentration further quantified using the Qubit high sensitivity RNA fluorometric assay (Thermo). Samples with RQN ≥9 were submitted to Novogene Corporation Inc for stranded mRNA library preparation with poly A enrichment and sequenced on an Illumina Novaseq 6000 with 150 bp paired end reads at 40–50 million reads per sample, with a final Q30 score of >90%. Raw data was filtered to remove adapters and low-quality reads. rRNA reads were filtered out using bowtie2 aligner (v2.3.5) with default settings. Reads were then aligned to the human hg38_gencode_V34 reference genome using STAR (v2.6.1) and count matrices were produced using FeatureCounts. Count data were analyzed for differential gene expression using DEBrowser and DESeq2, followed by gProfiler analysis of pathway enrichment. Corresponding plots and heat maps were produced in DEBrowser, R, and/or GraphPad Prism v8.

### Quantitative PCR (qPCR).

For samples from HNF4α^ΔIEC^ mice, RNA was reverse transcribed using iScript cDNA synthesis kit (Bio-Rad). For all other experiments, RNA was reverse transcribed into cDNA using the Quantitect Reverse Transcription assay (Qiagen). mRNA expression was quantified using TaqMan probe by qPCR using the ViiA7 real-time PCR system (ThermoFisher), using the following assays:[Table tab1]

**Table tab1:** 

Gene	TaqMan assay ID (ThermoFisher)
Human Abcb1	Hs00184500_m1
Human ARNTL	Hs00154147_m1
Human CEBPB	Hs00270923_s1
Human Cyp24A1	Hs00167999_m1
Human Cyp3A4	Hs00604506_m1
Human FOS	Hs00170630_m1
Human GAPDH	Hs02758991_g1
Human HIF1a	Hs00153153_m1
Human HMOX1	Hs01110250_m1
Human HNF4A	Hs00230853_m1
Human JUN	Hs99999141_s1
Human NFE2L2	Hs00975961_g1
Human NFKB1	Hs00765730_m1
Human Nqo1	Hs00168547_m1
Human Pim1	Hs01065498_m1
Mouse Abcb1a	Mm00440761_m1
Mouse ARNTL	Mm00500223_m1
Mouse HPRT	Mm03024075_m1

ΔCt values were calculated using GAPDH (human) or HPRT (mouse) and ΔΔC_t_ values were used for statistical analysis of differential expression. Data are reported as fold change (2^(-ΔΔCt)^) relative gene expression.

### Lysate generation.

Whole cell lysates of T84 and Caco2 cells were prepared for Western blot as previous described (23). Briefly, cells were washed in PBS then lysed at 4°C in buffer (20 mM Tris pH 7.4, 120 mM NaCl, 1 mM EDTA, 1% Triton X-100, 0.5% sodium deoxycholate, 1x protease inhibitor cocktail [Roche]) for 30 min followed by centrifugation at 13,000rpm for 5 min at 4°C. Cleared lysates were stored at −20°C until further analysis.

Nuclear and cytoplasmic fractions of cell lysates were prepared as previously described by Rockland Inc. (85). Briefly, cells were washed in PBS prior to cytoplasmic extract (CE) collection in CE buffer (10 mM HEPES, 60 mM KCl, 1 mM EDTA, 0.075% (vol/vol) NP-40, 1 mM DTT, 1 mM PMSF, pH 7.6). Remaining nuclear pellets were lysed in nuclear extract buffer (NE) (20 mM Tris Cl, 420 mM NaCl, 1.5 mM MgCl_2_, 0.2 mM EDTA, 1 mM PMSF, and 25% (vol/vol) glycerol, pH 8.0). CE and NE extracts were centrifuged at 29,000g for 10 min at 4°C and cleared lysates supplemented with glycerol were stored at −80°C.

### Western blot.

Protein concentration in cleared lysates was measured using the DC Assay (Bio-Rad). Samples were prepared for SDS-PAGE using LDS sample buffer (ThermoFisher) supplemented with 12.5 mM DTT before heating at 70°C for 10 min. Samples were loaded onto NuPAGE 3 to 8% Tris Acetate (ThermoFisher) or 4 to 20% Tris glycine (Bio-Rad) gels and electrophoresis run at 120V. Samples were transferred to nitrocellulose prior to blocking in Intercept PBS Blocking Buffer (LI-COR). Primary antibodies [anti-Pgp clone C219 (Millipore) at 1:500, anti-NRF2 (Abcam cat# Ab137550) at 1:4,000, anti-GAPDH (Millipore cat# MAB374) at 1:40,000, anti-Histone H3 (Cell Signaling cat# 14269) at 1:5,000, anti-H3K27Ac (Cell Signaling cat# 8173) at 1:500] were incubated on membranes overnight at 4°C. After washing with PBST (PBS + 0.1% Tween20), secondary antibodies (IRDye 800CW Goat anti-Mouse IgG, IRDye 800CW Goat anti-Rabbit IgG, IRDye 680RD Goat anti-Mouse IgG or IRDye 680RD Goat anti-Rabbit IgG) (LI-COR) at 1:5,000-1:200,000 were incubated protected from light for 1 h at room temperature prior to imaging on the Odyssey CLx (LI-COR). Densitometry was measured using Image Studio version 5.2 (LI-COR) with values normalized to internal protein loading control GAPDH.

### shRNA knockdown of NRF2.

shRNA knockdown of NRF2 in T84 cells was performed using lentiviral introduction of shRNA-expressing plasmid, similar to that previously described for T84 cells (1). Plasmids containing puromycin and ampicillin resistance genes and doxycycline-inducible promoters upstream of scrambled control shRNA (Tet-pLKO-puro-Scrambled, Addgene #47541), NRF2-targeting shRNA #1 (tet_pLKO.1_puro_shNRF2 #1, Addgene #136584), and NRF2-targeting shRNA #2 (tet_pLKO.1_puro_shNRF2 #2, Addgene #136585) were purchased from Addgene. These constructs were previously deposited by (86). Cultures from bacterial stabs were grown in Luria Broth (LB) with 100 μg/mL ampicillin prior to plasmid purification using the ZymoPURE II Plasmid Midiprep Kit (Zymo Research). Lentiviruses were produced by transfecting HEK293 cells with psPAX2, pMD.2G, and each pLKO.1 plasmid construct using *Trans*IT-293 transfection reagent (Mirius). After 48 and 72 h, lentiviral supernatants were harvested, pooled, and filtered through 0.45 μm low protein-binding membrane. T84 cells at 50% confluence were transduced with filtered lentivirus twice prior to selection using 10 μg/mL, 5 μg/mL, and 2 μg/mL puromycin in T84 growth media. Non-transduced T84 cells were incubated with puromycin as a control for selection. shRNA expression was induced by incubating cells with 100 μg/mL doxycycline (Sigma) in T84 growth media for 3 days. Knockdown of NRF2 expression was confirmed by qPCR and Western blot. To visualize NRF2 protein expression by Western blot, cells were first incubated with the proteasome inhibitor MG132 (Sigma) at 10 μM in T84 growth media for 6 h prior to lysate collection.

### CRISPR-Cas9 knockout of PXR.

Guide RNA (gRNA) sequences were selected based on CHOP CHOP (87–89) and CRISPick (Broad Institute) (90, 91) predictions, as well as reported gRNA sequences in the Toronto Knockout version 3.0 (TKOv3) library (92).

Single guide RNA (sgRNA) construct (gRNA sequence: CCAAATCTGCCGTGTATGTG), Cas9 nuclease, and electroporation enhancer were purchased from Integrated DNA Technologies (IDT). Ribonucleoprotein (RNP) complexes were prepared by incubation for 20 min at room temperature. RNPs were electroporated into T84 cells in suspension in Hanks Buffered Saline Solution (without Ca^2+^ or Mg^2+^) by 1 pulse at 1200V using GenePulser (Bio-Rad) in 0.4 cm cuvettes. Pooled cells were recovered for 5–7 days in growth media prior to single cell plating in 384-well tissue culture plates in growth media.

Genomic DNA was isolated from cells in suspension after washing with PBS using the Wizard Genomic DNA purification kit (Promega). DNA regions were amplified using 500units/reaction Q5 Hot start high fidelity polymerase (NEB), 200uM dNTP solution mix (NEB), 1uM primer (Forward primer: 5′-CACATGTTCTACTCCAGGGCTC-3′; Reverse primer: 5′-GGGTGAAGGCTGATGGGTAAC-3′), 1x Q5 reaction buffer, and 10 ng template DNA with thermalcycler conditions as follows: 98°C 30 s, 30 cycles of [98°C 10 s, annealing at 68°C 30 s, extension 72°C 30 s], 72°C 2 min. PCR amplicons were excised and purified using Gel Extraction Kit (Qiagen) and submitted for sanger sequencing to Azenta Life Sciences. Percentage of insertion/deletion (indel) were confirmed via ICE analysis (Synthego).

Single cell clones of T84 cells were expanded, gDNA indel confirmed as described above, and seeded for mRNA and protein analysis by qPCR and Western blot, respectively, as described above. Cells electroporated with Cas9 only were maintained as a control.

### Animal studies.

Mice were maintained in a specific pathogen-free (SPF) facility at the University of Massachusetts Chan Medical School.

**Circadian rhythm animal study.** Female C57BL/6J wild-type mice were purchased from Jackson laboratories (Bar Harbor, ME) and provided with irradiated standard chow (Prolab IsoPro #5P76) and acidified water *ad libitum*. Animals were housed 4 weeks to acclimate to the facility and light/dark cycle prior to beginning experiment. 8 week old animals were treated for 7 days with 0.5g/L cefoperazone (Sigma) in drinking water or plain water as vehicle control as previously described (23). Mice were euthanized every 4 h throughout a continuous 24-h cycle. Following euthanasia, colons were excised, rinsed in HBSS, and flash-frozen as 0.5 cm pieces for further analysis of RNA expression. For analysis of effect of antibiotic treatment on circadian rhythm-driven P-gp expression, amplitude of *Abcb1* for untreated and antibiotic-treated mice was calculated as (peak/trough) of the 24-h cycle as described in (93). Experiments were approved by the University of Massachusetts Chan Medical School Institutional Animal Care Use Committees (Protocol 202000132).

**HNF4α IEC conditional knockout mice.** *Hnf4a^f/f^* mice (94) were kindly gifted by Dr. Frank Gonzalez and crossed to *Vil-Cre* (Jax-021504). *Hnf4a^f/f^ Vil-Cre* were bred to *Hnf4a^f/f^* mice to generate experimental mice. *Vil-Cre*, *loxp* alleles and *Hnf4a* deletion was determined through Transnetyx. *Vil-Cre* positive and negative littermates remained cohoused after weaning. Both male and female mice were used for experiments at 8–12 weeks old. Following euthanization, whole colons were excised for intestinal epithelial cell isolation or 0.5 cm pieces of distal colon tissue were collected for further analysis of RNA expression. Experiments were approved by the Institutional Animal Care Use Committees (docket # A-1633-19).

**RNA isolation from mouse tissues.** For mouse samples from circadian rhythm experiments, flash-frozen mouse tissues were thawed directly into Buffer RLT (Qiagen) prior to homogenization using Lysing Matrix D (MP Biomedical). Total RNA from tissue was extracted using the RNeasy minikit (Qiagen) as described above for human cell lines. For samples from HNF4α^ΔIEC^ mice, Total RNA was extracted using Aurum Total RNA minikit (Bio-Rad). Colon tissue pieces (~0.5 cm) were sonicated in lysis buffer and processed per manufacture’s instruction. RNA concentrations were quantified by SpectraMax iD5 or NanoDrop. 500 ng of RNA was reversed transcribed using iScript cDNA synthesis kit (Bio-Rad). qPCR analysis of gene expression was performed as described above.

**Preparation of intestinal epithelial cell lysates.** Whole colon (between cecum and anus) was used. Intestines were opened longitudinally and washed with PBS to remove fecal contents. Tissues were cut into ~1 cm pieces and incubated in dissociation buffer (HBSS supplemented with 2.5 mM EDTA, 1 mM HEPES, 1 mM dithiothreitol (DTT), and 5% of FCS) at 37°C, 250 rpm for 30 min. Cells that passed through 70 μm cell strainers were washed with PBS and collected as IECs. IECs were resuspended in RIPA lysis buffer (ThermoFisher) supplemented with Halt protease inhibitor cocktail (ThermoFisher). Lysates were incubated on ice for 30 min, sonicated, and centrifuged to remove the debris.

### Data availability.

Access to databases and protocols generated under the project will be available for educational, research, and non-profit purposes. RNA sequencing data are accessible through NCBI GEO accession number GSE198478.

## References

[1] Szabady RL, Louissaint C, Lubben A, Xie B, Reeksting S, Tuohy C, Demma Z, Foley SE, Faherty CS, Llanos-Chea A, Olive AJ, Mrsny RJ, McCormick BA. 2018. Intestinal P-glycoprotein exports endocannabinoids to prevent inflammation and maintain homeostasis. J Clin Invest 128: 4044–4056. doi:10.1172/JCI96817. 30102254 PMC6118593

[2] Gottesman MM, Pastan I, Ambudkar SV. 1996. P-glycoprotein and multidrug resistance. Curr Opin Genet Dev 6:610–617. doi:10.1016/s0959-437x(96)80091-8. 8939727

[3] Ho GT, Aird RE, Liu B, Boyapati RK, Kennedy NA, Dorward DA, Noble CL, Shimizu T, Carter RN, Chew ETS, Morton NM, Rossi AG, Sartor RB, Iredale JP, Satsangi J. 2018. MDR1 deficiency impairs mitochondrial homeostasis and promotes intestinal inflammation. Mucosal Immunol 11:120–130. doi:10.1038/mi.2017.31. 28401939 PMC5510721

[4] Sharom FJ. 2011. The P-glycoprotein multidrug transporter. Essays Biochem 50:161–178. doi:10.1042/bse0500161. 21967057

[5] Blokzijl H, Vander Borght S, Bok LI, Libbrecht L, Geuken M, van den Heuvel FA, Dijkstra G, Roskams TA, Moshage H, Jansen PL, Faber KN. 2007. Decreased P-glycoprotein (P-gp/MDR1) expression in inflamed human intestinal epithelium is independent of PXR protein levels. Inflamm Bowel Dis 13:710–720. doi:10.1002/ibd.20088. 17262809

[6] Resta-Lenert S, Smitham J, Barrett KE. 2005. Epithelial dysfunction associated with the development of colitis in conventionally housed mdr1a−/− mice. Am J Physiol Gastrointest Liver Physiol 289:G153–62. doi:10.1152/ajpgi.00395.2004. 15774938

[7] Brinar M, Cukovic-Cavka S, Bozina N, Ravic KG, Markos P, Ladic A, Cota M, Krznaric Z, Vucelic B. 2013. MDR1 polymorphisms are associated with inflammatory bowel disease in a cohort of Croatian IBD patients. BMC Gastroenterol 13:1–7. doi:10.1186/1471-230X-13-57. 23537364 PMC3616873

[8] Brant SR, Panhuysen CI, Nicolae D, Reddy DM, Bonen DK, Karaliukas R, Zhang L, Swanson E, Datta LW, Moran T, Ravenhill G, Duerr RH, Achkar JP, Karban AS, Cho JH. 2003. MDR1 Ala893 polymorphism is associated with inflammatory bowel disease. Am J Hum Genet 73:1282–1292. doi:10.1086/379927. 14610718 PMC1180394

[9] Ho GT, Soranzo N, Nimmo ER, Tenesa A, Goldstein DB, Satsangi J. 2006. ABCB1/MDR1 gene determines susceptibility and phenotype in ulcerative colitis: discrimination of critical variants using a gene-wide haplotype tagging approach. Hum Mol Genet 15:797–805. doi:10.1093/hmg/ddi494. 16434479

[10] Panwala CM, Jones JC, Viney JL. 1998. A novel model of inflammatory bowel disease: mice deficient for the multiple drug resistance gene, mdr1a, spontaneously develop colitis. J Immunol 161:5733–5744. 9820555

[11] Wilk JN, Bilsborough J, Viney JL. 2005. The mdr1a−/− mouse model of spontaneous colitis. Immunol Res 31:151–159. doi:10.1385/IR:31:2:151. 15778512

[12] Chu XY, Strauss JR, Mariano MA, Li J, Newton DJ, Cai X, Wang RW, Yabut J, Hartley DP, Evans DC, Evers R. 2006. Characterization of mice lacking the multidrug resistance protein MRP2 (ABCC2). J Pharmacol Exp Ther 317:579–589. doi:10.1124/jpet.105.098665. 16421286

[13] Zaher H, Khan AA, Palandra J, Brayman TG, Yu L, Ware JA. 2006. Breast cancer resistance protein (Bcrp/abcg2) is a major determinant of sulfasalazine absorption and elimination in the mouse. Mol Pharm 3:55–61. doi:10.1021/mp050113v. 16686369

[14] Kim SN, Kim NH, Lee W, Seo DW, Kim YK. 2009. Histone deacetylase inhibitor induction of P-glycoprotein transcription requires both histone deacetylase 1 dissociation and recruitment of CAAT/enhancer binding protein beta and pCAF to the promoter region. Mol Cancer Res 7:735–744. doi:10.1158/1541-7786.MCR-08-0296. 19435809

[15] Scotto KW. 2003. Transcriptional regulation of ABC drug transporters. Oncogene 22:7496–7511. doi:10.1038/sj.onc.1206950. 14576854

[16] Silva R, Vilas-Boas V, Carmo H, Dinis-Oliveira RJ, Carvalho F, de Lourdes Bastos M, Remiao F. 2015. Modulation of P-glycoprotein efflux pump: induction and activation as a therapeutic strategy. Pharmacol Ther 149:1–123. doi:10.1016/j.pharmthera.2014.11.013. 25435018

[17] Hu Z, Jin S, Scotto KW. 2000. Transcriptional activation of the MDR1 gene by UV irradiation. Role of NF-Y and Sp1. J Biol Chem 275:2979–2985. doi:10.1074/jbc.275.4.2979. 10644769

[18] Chen Q, Bian Y, Zeng S. 2014. Involvement of AP-1 and NF-kappaB in the up-regulation of P-gp in vinblastine resistant Caco-2 cells. Drug Metab Pharmacokinet 29:223–226. doi:10.2133/dmpk.dmpk-13-sh-068. 24088727

[19] Chaudhary PM, Roninson IB. 1993. Induction of multidrug resistance in human cells by transient exposure to different chemotherapeutic drugs. J Natl Cancer Inst 85:632–639. doi:10.1093/jnci/85.8.632. 8096875

[20] Katayama K, Noguchi K, Sugimoto Y. 2014. Regulations of P-glycoprotein/ABCB1/MDR1in human cancer cells. New J Science 2014:1–10. doi:10.1155/2014/476974.

[21] Xie Y, Burcu M, Linn DE, Qiu Y, Baer MR. 2010. Pim-1 kinase protects P-glycoprotein from degradation and enables its glycosylation and cell surface expression. Mol Pharmacol 78:310–318. doi:10.1124/mol.109.061713. 20460432 PMC11037423

[22] Chambers TC, Pohl J, Glass DB, Kuo JF. 1994. Phosphorylation by protein kinase C and cyclic AMP-dependent protein kinase of synthetic peptides derived from the linker region of human P-glycoprotein. Biochem J 299:309–315. doi:10.1042/bj2990309. 7909431 PMC1138056

[23] Foley SE, Tuohy C, Dunford M, Grey MJ, De Luca H, Cawley C, Szabady RL, Maldonado-Contreras A, Houghton JM, Ward DV, Mrsny RJ, McCormick BA. 2021. Gut microbiota regulation of P-glycoprotein in the intestinal epithelium in maintenance of homeostasis. Microbiome 9:1–17. doi:10.1186/s40168-021-01137-3. 34493329 PMC8425172

[24] Mickley LA, Bates SE, Richert ND, Currier S, Tanaka S, Foss F, Rosen N, Fojo AT. 1989. Modulation of the expression of a multidrug resistance gene (mdr-1/P-glycoprotein) by differentiating agents. J Biol Chem 264:18031–18040. doi:10.1016/S0021-9258(19)84675-6. 2572588

[25] Furusawa Y, Obata Y, Fukuda S, Endo TA, Nakato G, Takahashi D, Nakanishi Y, Uetake C, Kato K, Kato T, Takahashi M, Fukuda NN, Murakami S, Miyauchi E, Hino S, Atarashi K, Onawa S, Fujimura Y, Lockett T, Clarke JM, Topping DL, Tomita M, Hori S, Ohara O, Morita T, Koseki H, Kikuchi J, Honda K, Hase K, Ohno H. 2013. Commensal microbe-derived butyrate induces the differentiation of colonic regulatory T cells. Nature 504:446–450. doi:10.1038/nature12721. 24226770

[26] Smith PM, Howitt MR, Panikov N, Michaud M, Gallini CA, Bohlooly-Y M, Glickman JN, Garrett WS. 2013. The microbial metabolites, short-chain fatty acids, regulate colonic treg cell homeostasis. Science 341:569–573. doi:10.1126/science.1241165. 23828891 PMC3807819

[27] Kelly CJ, Zheng L, Campbell EL, Saeedi B, Scholz CC, Bayless AJ, Wilson KE, Glover LE, Kominsky DJ, Magnuson A, Weir TL, Ehrentraut SF, Pickel C, Kuhn KA, Lanis JM, Nguyen V, Taylor CT, Colgan SP. 2015. Crosstalk between microbiota-derived short-chain fatty acids and intestinal epithelial HIF augments tissue barrier function. Cell Host Microbe 17:662–671. doi:10.1016/j.chom.2015.03.005. 25865369 PMC4433427

[28] Hamer HM, Jonkers D, Venema K, Vanhoutvin S, Troost FJ, Brummer RJ. 2008. Review article: the role of butyrate on colonic function. Aliment Pharmacol Ther 27:104–119. doi:10.1111/j.1365-2036.2007.03562.x. 17973645

[29] Thangaraju M, Cresci GA, Liu K, Ananth S, Gnanaprakasam JP, Browning DD, Mellinger JD, Smith SB, Digby GJ, Lambert NA, Prasad PD, Ganapathy V. 2009. GPR109A is a G-protein-coupled receptor for the bacterial fermentation product butyrate and functions as a tumor suppressor in colon. Cancer Res 69:2826–2832. doi:10.1158/0008-5472.CAN-08-4466. 19276343 PMC3747834

[30] Singh N, Gurav A, Sivaprakasam S, Brady E, Padia R, Shi H, Thangaraju M, Prasad PD, Manicassamy S, Munn DH, Lee JR, Offermanns S, Ganapathy V. 2014. Activation of Gpr109a, receptor for niacin and the commensal metabolite butyrate, suppresses colonic inflammation and carcinogenesis. Immunity 40:128–139. doi:10.1016/j.immuni.2013.12.007. 24412617 PMC4305274

[31] Kim MH, Kang SG, Park JH, Yanagisawa M, Kim CH. 2013. Short-chain fatty acids activate GPR41 and GPR43 on intestinal epithelial cells to promote inflammatory responses in mice. Gastroenterology 145:396–406. doi:10.1053/j.gastro.2013.04.056. 23665276

[32] Kim SY, Chae CW, Lee HJ, Jung YH, Choi GE, Kim JS, Lim JR, Lee JE, Cho JH, Park H, Park C, Han HJ. 2020. Sodium butyrate inhibits high cholesterol-induced neuronal amyloidogenesis by modulating NRF2 stabilization-mediated ROS levels: involvement of NOX2 and SOD1. Cell Death Dis 11:1–19. doi:10.1038/s41419-020-2663-1. 32555166 PMC7303181

[33] Dong W, Jia Y, Liu X, Zhang H, Li T, Huang W, Chen X, Wang F, Sun W, Wu H. 2017. Sodium butyrate activates NRF2 to ameliorate diabetic nephropathy possibly via inhibition of HDAC. J Endocrinol 232:71–83. doi:10.1530/JOE-16-0322. 27799462

[34] Davie JR. 2003. Inhibition of histone deacetylase activity by butyrate. J Nutr 133:2485S–2493S. doi:10.1093/jn/133.7.2485S. 12840228

[35] Dashwood RH, Myzak MC, Ho E. 2006. Dietary HDAC inhibitors: time to rethink weak ligands in cancer chemoprevention? Carcinogenesis 27:344–349. doi:10.1093/carcin/bgi253. 16267097 PMC2267878

[36] Sekhavat A, Sun JM, Davie JR. 2007. Competitive inhibition of histone deacetylase activity by trichostatin A and butyrate. Biochem Cell Biol 85:751–758. doi:10.1139/o07-145. 18059533

[37] Jin S, Scotto KW. 1998. Transcriptional regulation of the MDR1 gene by histone acetyltransferase and deacetylase is mediated by NF-Y. Mol Cell Biol 18:4377–4384. doi:10.1128/MCB.18.7.4377. 9632821 PMC109021

[38] Ridlon JM, Kang DJ, Hylemon PB. 2006. Bile salt biotransformations by human intestinal bacteria. J Lipid Res 47:241–259. doi:10.1194/jlr.R500013-JLR200. 16299351

[39] Devlin AS, Fischbach MA. 2015. A biosynthetic pathway for a prominent class of microbiota-derived bile acids. Nat Chem Biol 11:685–690. doi:10.1038/nchembio.1864. 26192599 PMC4543561

[40] Sinha SR, Haileselassie Y, Nguyen LP, Tropini C, Wang M, Becker LS, Sim D, Jarr K, Spear ET, Singh G, Namkoong H, Bittinger K, Fischbach MA, Sonnenburg JL, Habtezion A. 2020. Dysbiosis-induced secondary bile acid deficiency promotes intestinal inflammation. Cell Host Microbe 27:659–670. doi:10.1016/j.chom.2020.01.021. 32101703 PMC8172352

[41] Ward JBJ, Lajczak NK, Kelly OB, O'Dwyer AM, Giddam AK, Ni Gabhann J, Franco P, Tambuwala MM, Jefferies CA, Keely S, Roda A, Keely SJ. 2017. Ursodeoxycholic acid and lithocholic acid exert anti-inflammatory actions in the colon. Am J Physiol Gastrointest Liver Physiol 312:G550–G558. doi:10.1152/ajpgi.00256.2016. 28360029

[42] Buffie CG, Bucci V, Stein RR, McKenney PT, Ling L, Gobourne A, No D, Liu H, Kinnebrew M, Viale A, Littmann E, van den Brink MR, Jenq RR, Taur Y, Sander C, Cross JR, Toussaint NC, Xavier JB, Pamer EG. 2015. Precision microbiome reconstitution restores bile acid mediated resistance to Clostridium difficile. Nature 517:205–208. doi:10.1038/nature13828. 25337874 PMC4354891

[43] Ridlon JM, Bajaj JS. 2015. The human gut sterolbiome: bile acid-microbiome endocrine aspects and therapeutics. Acta Pharm Sin B 5:99–105. doi:10.1016/j.apsb.2015.01.006. 26579434 PMC4629220

[44] Shah YM, Ma X, Morimura K, Kim I, Gonzalez FJ. 2007. Pregnane X receptor activation ameliorates DSS-induced inflammatory bowel disease via inhibition of NF-kappaB target gene expression. Am J Physiol Gastrointest Liver Physiol 292:G1114–G1122. doi:10.1152/ajpgi.00528.2006. 17170021

[45] White JH. 2018. Vitamin D deficiency and the pathogenesis of Crohn's disease. J Steroid Biochem Mol Biol 175:23–28. doi:10.1016/j.jsbmb.2016.12.015. 28025175

[46] Kong J, Zhang Z, Musch MW, Ning G, Sun J, Hart J, Bissonnette M, Li YC. 2008. Novel role of the vitamin D receptor in maintaining the integrity of the intestinal mucosal barrier. Am J Physiol Gastrointest Liver Physiol 294:G208–G216. doi:10.1152/ajpgi.00398.2007. 17962355

[47] Tachibana S, Yoshinari K, Chikada T, Toriyabe T, Nagata K, Yamazoe Y. 2009. Involvement of Vitamin D receptor in the intestinal induction of human ABCB1. Drug Metab Dispos 37:1604–1610. doi:10.1124/dmd.109.027219. 19460946

[48] Carazo A, Hyrsova L, Dusek J, Chodounska H, Horvatova A, Berka K, Bazgier V, Gan-Schreier H, Chamulitrat W, Kudova E, Pavek P. 2017. Acetylated deoxycholic (DCA) and cholic (CA) acids are potent ligands of pregnane X (PXR) receptor. Toxicol Lett 265:86–96. doi:10.1016/j.toxlet.2016.11.013. 27871908

[49] Stenman LK, Holma R, Eggert A, Korpela R. 2013. A novel mechanism for gut barrier dysfunction by dietary fat: epithelial disruption by hydrophobic bile acids. Am J Physiol Gastrointest Liver Physiol 304:G227–G234. doi:10.1152/ajpgi.00267.2012. 23203158

[50] Sarathy J, Detloff SJ, Ao M, Khan N, French S, Sirajuddin H, Nair T, Rao MC. 2017. The Yin and Yang of bile acid action on tight junctions in a model colonic epithelium. Physiol Rep 5:e13294. doi:10.14814/phy2.13294. 28554966 PMC5449568

[51] Makishima M, Okamoto AY, Repa JJ, Tu H, Learned RM, Luk A, Hull MV, Lustig KD, Mangelsdorf DJ, Shan B. 1999. Identification of a nuclear receptor for bile acids. Science 284:1362–1365. doi:10.1126/science.284.5418.1362. 10334992

[52] Wu S, Li RW, Li W, Li CJ. 2012. Transcriptome characterization by RNA-seq unravels the mechanisms of butyrate-induced epigenomic regulation in bovine cells. PLoS One 7:e36940. doi:10.1371/journal.pone.0036940. 22615851 PMC3352864

[53] Schulthess J, Pandey S, Capitani M, Rue-Albrecht KC, Arnold I, Franchini F, Chomka A, Ilott NE, Johnston DGW, Pires E, McCullagh J, Sansom SN, Arancibia-Carcamo CV, Uhlig HH, Powrie F. 2019. The short chain fatty acid butyrate imprints an antimicrobial program in macrophages. Immunity 50:432–445. doi:10.1016/j.immuni.2018.12.018. 30683619 PMC6382411

[54] Sundqvist A, Vasilaki E, Voytyuk O, Bai Y, Morikawa M, Moustakas A, Miyazono K, Heldin CH, Ten Dijke P, van Dam H. 2020. TGFbeta and EGF signaling orchestrates the AP-1- and p63 transcriptional regulation of breast cancer invasiveness. Oncogene 39:4436–4449. doi:10.1038/s41388-020-1299-z. 32350443 PMC7253358

[55] Priyamvada S, Anbazhagan AN, Kumar A, Soni V, Alrefai WA, Gill RK, Dudeja PK, Saksena S. 2016. Lactobacillus acidophilus stimulates intestinal P-glycoprotein expression via a c-Fos/c-Jun-dependent mechanism in intestinal epithelial cells. Am J Physiol Gastrointest Liver Physiol 310:G599–G608. doi:10.1152/ajpgi.00210.2015. 26867563 PMC4836133

[56] Kubo M. 2020. Diurnal rhythmicity programs of microbiota and transcriptional oscillation of circadian regulator, NFIL3. Front Immunol 11:552188. doi:10.3389/fimmu.2020.552188. 33013924 PMC7511535

[57] Zhang YK, Yeager RL, Klaassen CD. 2009. Circadian expression profiles of drug-processing genes and transcription factors in mouse liver. Drug Metab Dispos 37:106–115. doi:10.1124/dmd.108.024174. 18838502 PMC2683654

[58] Thaiss CA, Levy M, Korem T, Dohnalova L, Shapiro H, Jaitin DA, David E, Winter DR, Gury-BenAri M, Tatirovsky E, Tuganbaev T, Federici S, Zmora N, Zeevi D, Dori-Bachash M, Pevsner-Fischer M, Kartvelishvily E, Brandis A, Harmelin A, Shibolet O, Halpern Z, Honda K, Amit I, Segal E, Elinav E. 2016. Microbiota diurnal rhythmicity programs host transcriptome oscillations. Cell 167:1495–1510. doi:10.1016/j.cell.2016.11.003. 27912059

[59] Dulong S, Ballesta A, Okyar A, Levi F. 2015. Identification of circadian determinants of cancer chronotherapy through *in vitro* chronopharmacology and mathematical modeling. Mol Cancer Ther 14:2154–2164. doi:10.1158/1535-7163.MCT-15-0129. 26141947

[60] Murakami Y, Higashi Y, Matsunaga N, Koyanagi S, Ohdo S. 2008. Circadian clock-controlled intestinal expression of the multidrug-resistance gene mdr1a in mice. Gastroenterology 135:1636–1644. doi:10.1053/j.gastro.2008.07.073. 18773899

[61] Combates NJ, Rzepka RW, Chen YN, Cohen D. 1994. NF-IL6, a member of the C/EBP family of transcription factors, binds and trans-activates the human MDR1 gene promoter. J Biological Chemistry 269:29715–29719. doi:10.1016/S0021-9258(18)43939-7. 7961962

[62] Riganti C, Doublier S, Viarisio D, Miraglia E, Pescarmona G, Ghigo D, Bosia A. 2009. Artemisinin induces doxorubicin resistance in human colon cancer cells via calcium-dependent activation of HIF-1alpha and P-glycoprotein overexpression. Br J Pharmacol 156:1054–1066. doi:10.1111/j.1476-5381.2009.00117.x. 19298255 PMC2697684

[63] Riganti C, Campia I, Polimeni M, Pescarmona G, Ghigo D, Bosia A. 2009. Digoxin and ouabain induce P-glycoprotein by activating calmodulin kinase II and hypoxia-inducible factor-1alpha in human colon cancer cells. Toxicol Appl Pharmacol 240:385–392. doi:10.1016/j.taap.2009.07.026. 19647009

[64] Chen J, Ding Z, Peng Y, Pan F, Li J, Zou L, Zhang Y, Liang H. 2014. HIF-1alpha inhibition reverses multidrug resistance in colon cancer cells via downregulation of MDR1/P-glycoprotein. PLoS One 9:e98882. doi:10.1371/journal.pone.0098882. 24901645 PMC4047061

[65] Ogretmen B, Safa AR. 1999. Negative regulation of *MDR1* promoter activity in MCF-7, but not in multidrug resistant MCF-7/Adr, cells by cross-coupled NF-kB/p65 and c-Fos transcription factors and their interaction with the CAAT region. Biochemistry 38:2189–2199. doi:10.1021/bi982236+. 10026303

[66] Kamiyama Y, Matsubara T, Yoshinari K, Nagata K, Kamimura H, Yamazoe Y. 2007. Role of human hepatocyte nuclear factor 4a in the expression of drug-metabolizing enzymes and transporters in human hepatocytes assessed by use of small interfering RNA. Drug Metab Pharmacokinet 22:287–298. doi:10.2133/dmpk.22.287. 17827783

[67] Darsigny M, Babeu JP, Dupuis AA, Furth EE, Seidman EG, Levy E, Verdu EF, Gendron FP, Boudreau F. 2009. Loss of hepatocyte-nuclear-factor-4alpha affects colonic ion transport and causes chronic inflammation resembling inflammatory bowel disease in mice. PLoS One 4:e7609. doi:10.1371/journal.pone.0007609. 19898610 PMC2764139

[68] Nguyen T, Nioi P, Pickett CB. 2009. The Nrf2-antioxidant response element signaling pathway and its activation by oxidative stress. J Biol Chem 284:13291–13295. doi:10.1074/jbc.R900010200. 19182219 PMC2679427

[69] Jing W, Safarpour Y, Zhang T, Guo P, Chen G, Wu X, Fu Q, Wang Y. 2018. Berberine upregulates P-glycoprotein in human Caco-2 cells and in an experimental model of colitis in the rat via activation of Nrf2-dependent mechanisms. J Pharmacol Exp Ther 366:332–340. doi:10.1124/jpet.118.249615. 29891588

[70] Wang X, Campos CR, Peart JC, Smith LK, Boni JL, Cannon RE, Miller DS. 2014. Nrf2 upregulates ATP binding cassette transporter expression and activity at the blood-brain and blood-spinal cord barriers. J Neurosci 34:8585–8593. doi:10.1523/JNEUROSCI.2935-13.2014. 24948812 PMC4061395

[71] Foster SR, Blank K, See Hoe LE, Behrens M, Meyerhof W, Peart JN, Thomas WG. 2014. Bitter taste receptor agonists elicit G-protein-dependent negative inotropy in the murine heart. FASEB J 28:4497–4508. doi:10.1096/fj.14-256305. 25002118

[72] Ayoub MA, Damian M, Gespach C, Ferrandis E, Lavergne O, De Wever O, Baneres JL, Pin JP, Prevost GP. 2009. Inhibition of heterotrimeric G protein signaling by a small molecule acting on Galpha subunit. J Biol Chem 284:29136–29145. doi:10.1074/jbc.M109.042333. 19648112 PMC2781458

[73] Beautrait A, Paradis JS, Zimmerman B, Giubilaro J, Nikolajev L, Armando S, Kobayashi H, Yamani L, Namkung Y, Heydenreich FM, Khoury E, Audet M, Roux PP, Veprintsev DB, Laporte SA, Bouvier M. 2017. A new inhibitor of the beta-arrestin/AP2 endocytic complex reveals interplay between GPCR internalization and signalling. Nat Commun 8:15054. doi:10.1038/ncomms15054. 28416805 PMC5399295

[74] Kheir TB, Lund AH. 2010. Epigenetic dynamics across the cell cycle. Essays Biochem 48:107–120. doi:10.1042/bse0480107. 20822490

[75] Williams MS, Amaral FM, Simeoni F, Somervaille TC. 2020. A stress-responsive enhancer induces dynamic drug resistance in acute myeloid leukemia. J Clin Invest 130:1217–1232. doi:10.1172/JCI130809. 31770110 PMC7269587

[76] Langmann T, Moehle C, Mauerer R, Scharl M, Liebisch G, Zahn A, Stremmel W, Schmitz G. 2004. Loss of detoxification in inflammatory bowel disease: dysregulation of pregnane X receptor target genes. Gastroenterology 127:26–40. doi:10.1053/j.gastro.2004.04.019. 15236169

[77] Cascorbi I. 2006. Role of pharmacogenetics of ATP-binding cassette transporters in the pharmacokinetics of drugs. Pharmacol Ther 112:457–473. doi:10.1016/j.pharmthera.2006.04.009. 16766035

[78] Davison JM, Lickwar CR, Song L, Breton G, Crawford GE, Rawls JF. 2017. Microbiota regulate intestinal epithelial gene expression by suppressing the transcription factor Hepatocyte nuclear factor 4 alpha. Genome Res 27:1195–1206. doi:10.1101/gr.220111.116. 28385711 PMC5495071

[79] Rothschild D, Weissbrod O, Barkan E, Kurilshikov A, Korem T, Zeevi D, Costea PI, Godneva A, Kalka IN, Bar N, Shilo S, Lador D, Vila AV, Zmora N, Pevsner-Fischer M, Israeli D, Kosower N, Malka G, Wolf BC, Avnit-Sagi T, Lotan-Pompan M, Weinberger A, Halpern Z, Carmi S, Fu J, Wijmenga C, Zhernakova A, Elinav E, Segal E. 2018. Environment dominates over host genetics in shaping human gut microbiota. Nature 555:210–215. doi:10.1038/nature25973. 29489753

[80] Olendzki B, Bucci V, Cawley C, Maserati R, McManus M, Olednzki E, Madziar C, Chiang D, Ward DV, Pellish R, Foley C, Bhattarai S, McCormick BA, Maldonado-Contreras A. 2022. Dietary manipulation of the gut microbiome in inflammatory bowel disease patients: pilot study. Gut Microbes 14:2046244. doi:10.1080/19490976.2022.2046244. 35311458 PMC8942410

[81] Olsson LM, Boulund F, Nilsson S, Khan MT, Gummesson A, Fagerberg L, Engstrand L, Perkins R, Uhlen M, Bergstrom G, Tremaroli V, Backhed F. 2022. Dynamics of the normal gut microbiota: A longitudinal one-year population study in Sweden. Cell Host Microbe. doi:10.1016/j.chom.2022.03.002. 35349787

[82] Fornelos N, Franzosa EA, Bishai J, Annand JW, Oka A, Lloyd-Price J, Arthur TD, Garner A, Avila-Pacheco J, Haiser HJ, Tolonen AC, Porter JA, Clish CB, Sartor RB, Huttenhower C, Vlamakis H, Xavier RJ. 2020. Growth effects of N-acylethanolamines on gut bacteria reflect altered bacterial abundances in inflammatory bowel disease. Nat Microbiol 5:486–497. doi:10.1038/s41564-019-0655-7. 31959971 PMC7047597

[83] Lin L, Zhang J. 2017. Role of intestinal microbiota and metabolites on gut homeostasis and human diseases. BMC Immunol 18:2. doi:10.1186/s12865-016-0187-3. 28061847 PMC5219689

[84] Russell DW. 2003. The enzymes, regulation, and genetics of bile acid synthesis. Annu Rev Biochem 72:137–174. doi:10.1146/annurev.biochem.72.121801.161712. 12543708

[85] Baldwin AS. 1996. The NF-kB and IkB proteins: new discoveries and insights. Annu Rev Immunol 14:649–681. doi:10.1146/annurev.immunol.14.1.649. 8717528

[86] Pereira EJ, Burns JS, Lee CY, Marohl T, Calderon D, Wang L, Atkins KA, Wang C-C, Janes KA. 2020. Sporadic activation of an oxidative stress-dependent NRF2-p53 signaling network in breast epithelial spheroids and premalignancies. Sci Signal 13. doi:10.1126/scisignal.aba4200. PMC7315801 32291314

[87] Labun K, Montague TG, Krause M, Torres Cleuren YN, Tjeldnes H, Valen E. 2019. CHOPCHOP v3: expanding the CRISPR web toolbox beyond genome editing. Nucleic Acids Res 47:W171–W174. doi:10.1093/nar/gkz365. 31106371 PMC6602426

[88] Labun K, Montague TG, Gagnon JA, Thyme SB, Valen E. 2016. CHOPCHOP v2: a web tool for the next generation of CRISPR genome engineering. Nucleic Acids Res 44:W272–6. doi:10.1093/nar/gkw398. 27185894 PMC4987937

[89] Montague TG, Cruz JM, Gagnon JA, Church GM, Valen E. 2014. CHOPCHOP: a CRISPR/Cas9 and TALEN web tool for genome editing. Nucleic Acids Res 42:W401–7. doi:10.1093/nar/gku410. 24861617 PMC4086086

[90] Doench JG, Fusi N, Sullender M, Hegde M, Vaimberg EW, Donovan KF, Smith I, Tothova Z, Wilen C, Orchard R, Virgin HW, Listgarten J, Root DE. 2016. Optimized sgRNA design to maximize activity and minimize off-target effects of CRISPR-Cas9. Nat Biotechnol 34:184–191. doi:10.1038/nbt.3437. 26780180 PMC4744125

[91] Sanson KR, Hanna RE, Hegde M, Donovan KF, Strand C, Sullender ME, Vaimberg EW, Goodale A, Root DE, Piccioni F, Doench JG. 2018. Optimized libraries for CRISPR-Cas9 genetic screens with multiple modalities. Nat Commun 9:5416. doi:10.1038/s41467-018-07901-8. 30575746 PMC6303322

[92] Hart T, Tong AHY, Chan K, Van Leeuwen J, Seetharaman A, Aregger M, Chandrashekhar M, Hustedt N, Seth S, Noonan A, Habsid A, Sizova O, Nedyalkova L, Climie R, Tworzyanski L, Lawson K, Sartori MA, Alibeh S, Tieu D, Masud S, Mero P, Weiss A, Brown KR, Usaj M, Billmann M, Rahman M, Constanzo M, Myers CL, Andrews BJ, Boone C, Durocher D, Moffat J. 2017. Evaluation and design of genome-wide CRISPR/SpCas9 knockout screens. G3 (Bethesda) 7:2719–2727. doi:10.1534/g3.117.041277. 28655737 PMC5555476

[93] Prior KF, Rijo-Ferreira F, Assis PA, Hirako IC, Weaver DR, Gazzinelli RT, Reece SE. 2020. Periodic parasites and daily host rhythms. Cell Host Microbe 27:176–187. doi:10.1016/j.chom.2020.01.005. 32053788 PMC7137616

[94] Hayhurst GP, Lee YH, Lambert G, Ward JM, Gonzalez FJ. 2001. Hepatocyte nuclear factor 4alpha (nuclear receptor 2A1) is essential for maintenance of hepatic gene expression and lipid homeostasis. Mol Cell Biol 21:1393–1403. doi:10.1128/MCB.21.4.1393-1403.2001. 11158324 PMC99591

