# Supplementary figures and images for: Microbial Metabolites Orchestrate a Distinct Multi-Tiered Regulatory Network in the Intestinal Epithelium That Directs P-Glycoprotein Expression
Source: mBio. 2022 Aug 15;13(4):e01993-22. doi: 10.1128/mbio.01993-22 (PMC9426490; doi:10.1128/mbio.01993-22)

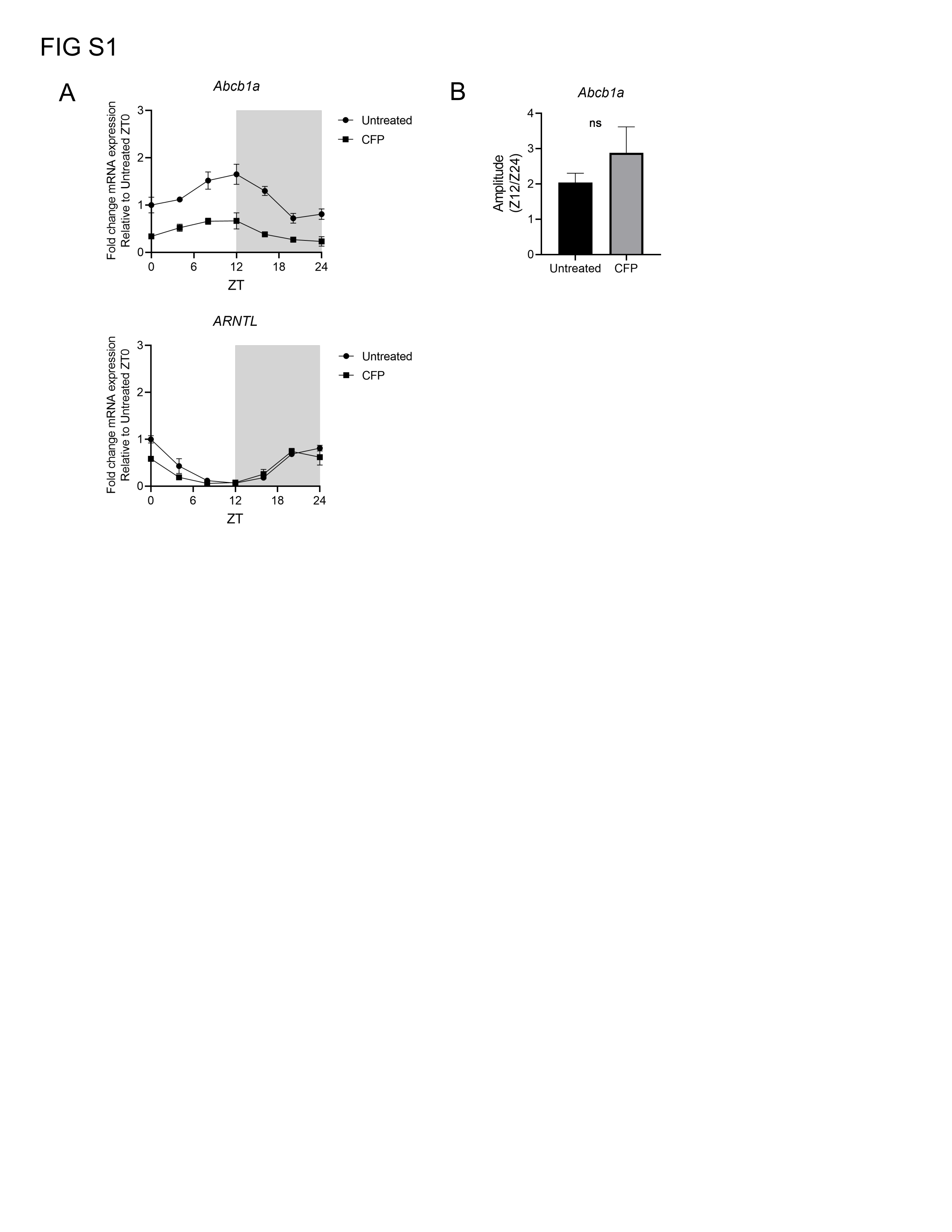

Supplement: FIG S1 [file mbio.01993-22-s0001.tif]

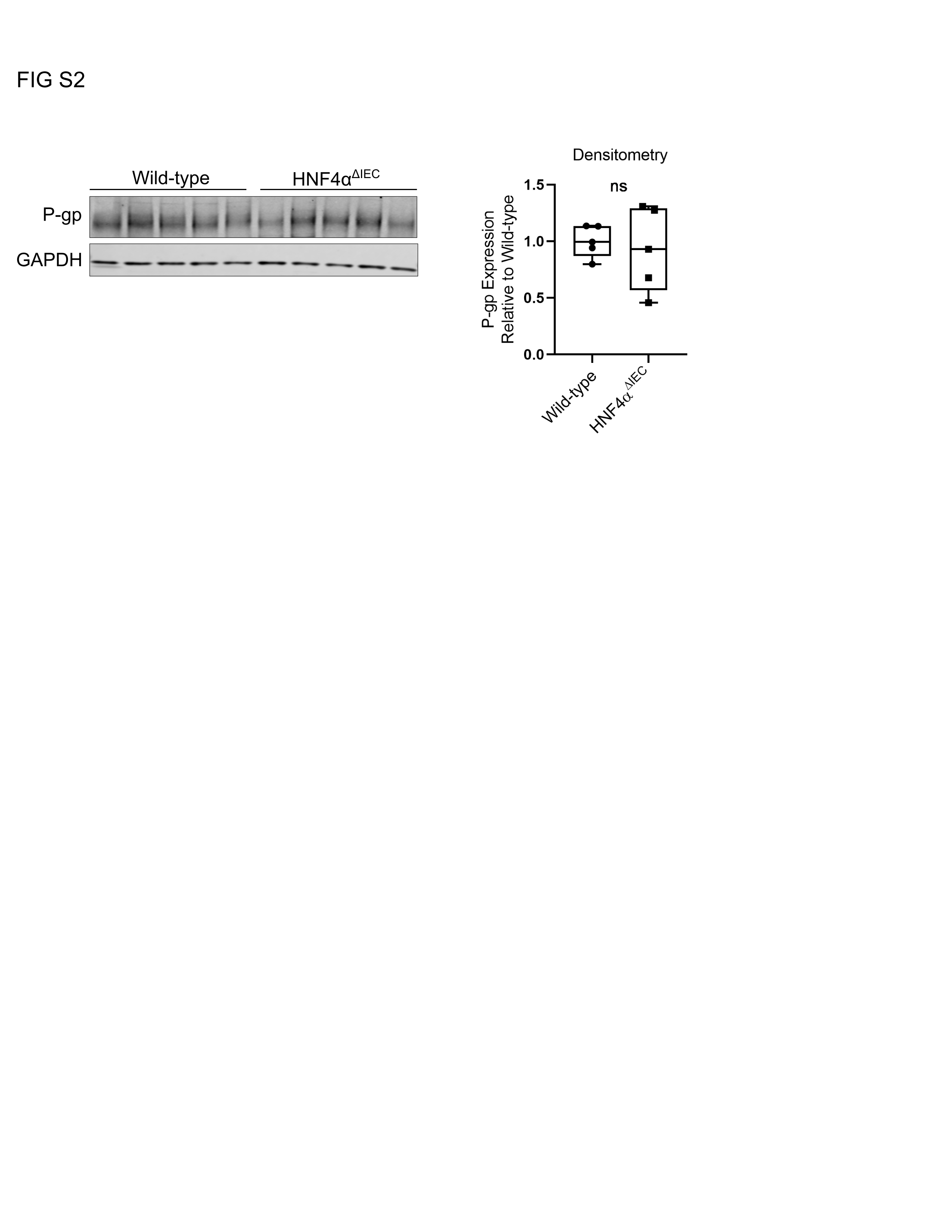

Supplement: FIG S2 [file mbio.01993-22-s0002.tif]

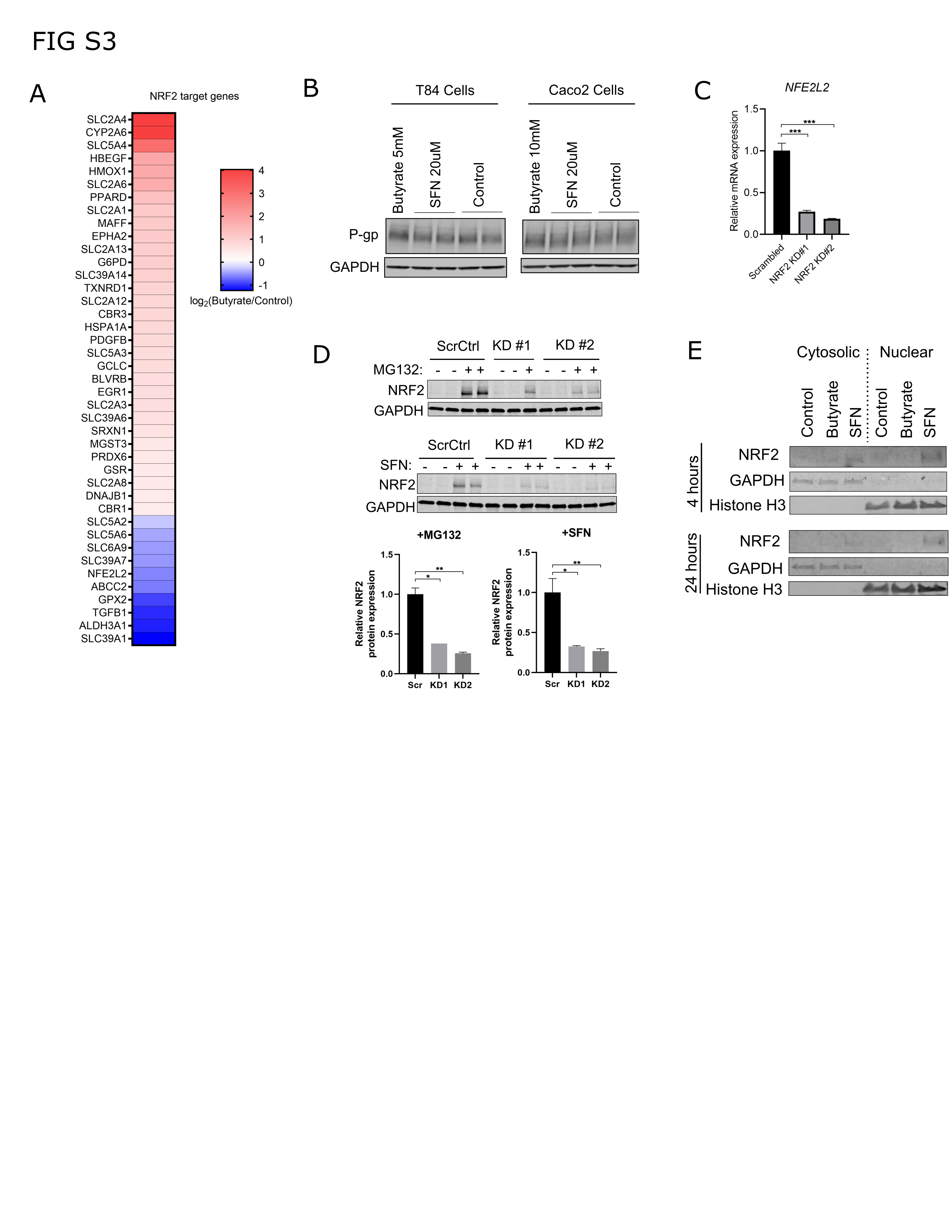

Supplement: FIG S3 [file mbio.01993-22-s0003.tif]

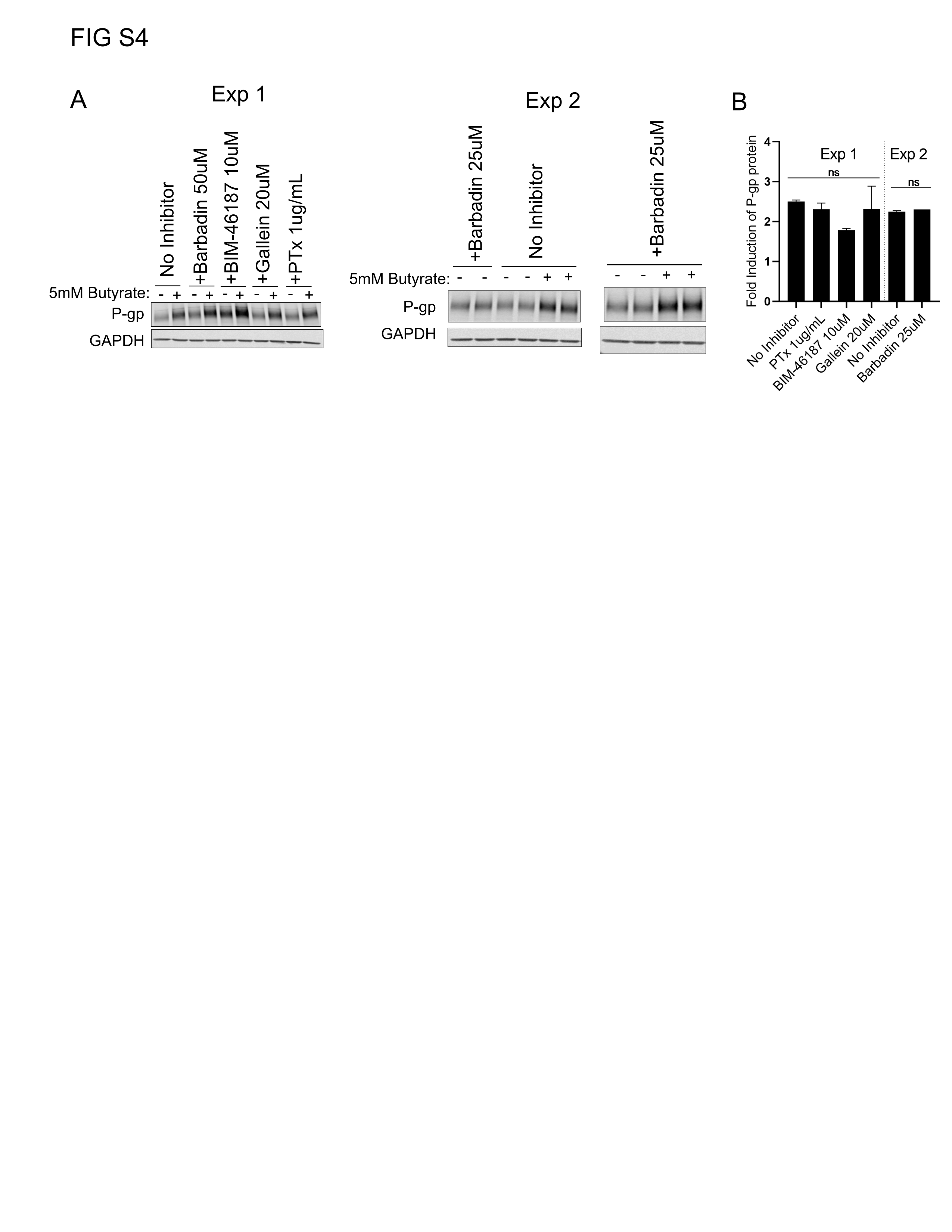

Supplement: FIG S4 [file mbio.01993-22-s0004.tif]

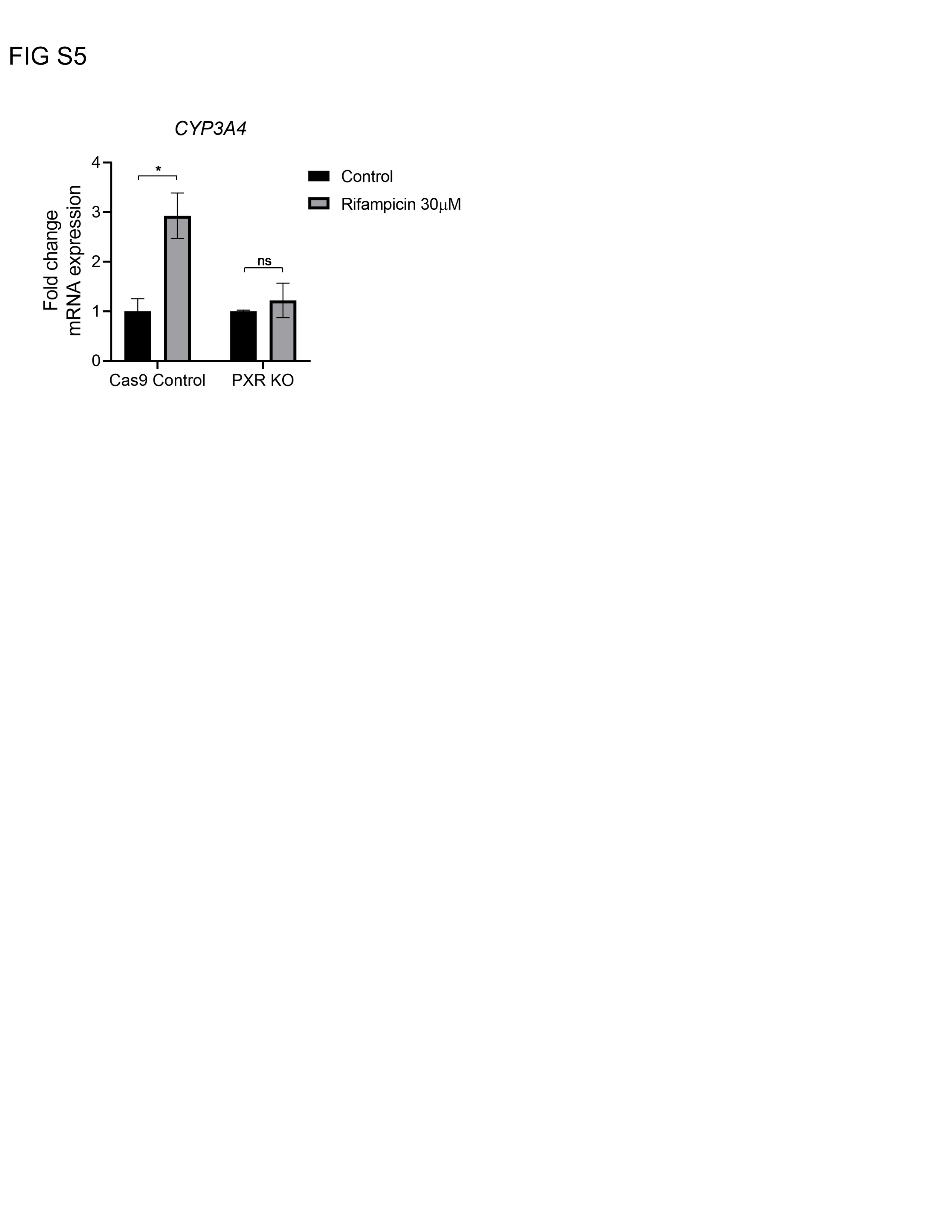

Supplement: FIG S5 [file mbio.01993-22-s0005.tif]
